# NB-LRR-encoding genes conferring susceptibility to organophosphate pesticides in sorghum

**DOI:** 10.1038/s41598-021-98908-7

**Published:** 2021-10-06

**Authors:** Zihuan Jing, Fiona Wacera W., Tsuneaki Takami, Hideki Takanashi, Fumi Fukada, Yoji Kawano, Hiromi Kajiya-Kanegae, Hiroyoshi Iwata, Nobuhiro Tsutsumi, Wataru Sakamoto

**Affiliations:** 1 Institute of Plant Science and Resources, Okayama University, 2-20-1 Chuo, Kurashiki, Okayama 710-0046 Japan; 2 Graduate School of Agricultural and Life Sciences, The University of Tokyo, 1-1-1 Yayoi, Bunkyo-ku, Tokyo 113-8657 Japan; 3 Research Center for Agricultural Information Technology, National Agriculture and Food Research Organization, Minato-ku, Tokyo 105-0003 Japan

**Keywords:** Plant breeding, Abiotic, Plant physiology

## Abstract

Organophosphate is the commonly used pesticide to control pest outbreak, such as those by aphids in many crops. Despite its wide use, however, necrotic lesion and/or cell death following the application of organophosphate pesticides has been reported to occur in several species. To understand this phenomenon, called organophosphate pesticide sensitivity (OPS) in sorghum, we conducted QTL analysis in a recombinant inbred line derived from the Japanese cultivar NOG, which exhibits OPS. Mapping OPS in this population identified a prominent QTL on chromosome 5, which corresponded to *Organophosphate-Sensitive Reaction* (*OSR*) reported previously in other mapping populations. The *OSR* locus included a cluster of three genes potentially encoding nucleotide-binding leucine-rich repeat (NB-LRR, NLR) proteins, among which *NLR-C* was considered to be responsible for OPS in a dominant fashion. *NLR-C* was functional in NOG, whereas the other resistant parent, BTx623, had a null mutation caused by the deletion of promoter sequences. Our finding of *OSR* as a dominant trait is important not only in understanding the diversified role of NB-LRR proteins in cereals but also in securing sorghum breeding free from OPS.

## Introduction

Crops growing under natural conditions face the threats from different environmental factors, among which pathogens and pests often cause a serious loss of yield^1–3^. To counteract such biotic stresses, breeding crops that confer resistance to them is demanding^4,5^. Plants are known to defense against pathogens by immune responses with *R* genes^6–8^. The majority of genetically characterized disease resistance traits map to *R* genes encoding nucleotide-binding domain (NB) and leucine-rich repeat (LRR) proteins, and they act as receptors to perceive effectors derived from pathogens and to activate effector-trigger immunity (ETI), which includes cell death and reactive oxygen species production^9–16^. In addition to harnessing these resistant genes, chemical management of pests is a practical way; plants are applied by the pesticides to manage the insects that attack a broad range of crop species^17,18^. Controlling aphids, thrips and other pests are important not only to avoid yield loss by severe infestation but also to prevent these insects from spreading vector-borne diseases^19–22^.

Organophosphates are a group of commonly used pesticides that kill insects by causing neurotoxicity through the inhibition acetylcholinesterase activity, whereas these compounds show no toxicity to host plants^23^. Cell death or leaf injury that resembles the hypersensitive reaction to pathogens, however, is rarely observed when crops are sprayed with a particular type of organophosphate pesticides^24^. Molecular analysis of such organophosphate pesticide sensitivity (OPS) was first reported in tomato; plants harboring the *Pto* locus (conferring resistance to *Pseudomonas syringae* pv. *tomato* strains) exhibited necrosis after being sprayed with fenthion, an organophosphate pesticide^25–27^. This OPS phenotype in tomato was shown to depend on the *Fen* gene, encoding a serine-threonine protein kinase belonging to the receptor-like kinases among the *R* genes^28^. More recently, another nucleotide-binding leucine-rich repeat (NB-LRR) gene termed *Prf* embedded within the *Pto/Fen* gene cluster was shown to act in concert with *Pto/Fen* to activate multiple plant–pathogen signal transduction pathways^29–31^.

The other example of NB-LRR responding to organic chemicals is VICTR in *Arabidopsis*, which responds to accession-specific root growth arrest with the small-molecule [5-(3,4-dichlorophenyl) furan-2-yl]-piperidine-1-ylmethanethione (DFPM)^32,33^. These findings present the intriguing recognition mechanism of external chemicals through NB-LRR, whereas the cases are limited to several species. In this study, we focused on another example of OPS in sorghum, which has segregated in a recombinant inbred population we recently established.

Sorghum (*Sorghum bicolor* L. Moench), is the fifth most important cereal crop worldwide^34^, and its high biomass along with extreme tolerance to drought and high-temperature conditions makes sorghum a potential crop for food, fodder, and bioenergy production^35^. Like other crops, sorghum is often devastated by aphid infestations and the organophosphate pesticides are routinely used. During the sorghum growing period from May to August that includes rainy season in Japan, an outbreak of the major aphid *Melanaphis sacchari* (Zehntner) occurs in our test field and elsewhere in Japan^36,37^. Its reproductive capacity is extremely high, and the plants result in turning leaves into rusty brown and necrosis, eventually leading to plant death^38^. In order to control the aphid population effectively, we use organophosphate pesticides, some of which were then shown to cause OPS and result in brown spotting lesion both in leaves and stems. This OPS appeared to derive from one Japanese cultivar NOG, whose generic name is Takakibi (alternatively called ‘Morokoshi’ or ‘Koryan’, the latter is the Chinese sorghum named ‘Gaoliang’ in Japanese).

To investigate OPS in sorghum further, we utilized a recombinant inbred line (RIL) population, generated by a cross between BTx623 and NOG^39,40^. Our previous studies indicated that NOG and BTx623 differ not only in morphological traits such as days to heading and plant height but also in photosynthetic response to high temperature and strong light around the heading period^39,41^. The development of genetic map with high-density markers (> 3700), generated by RAD-seq in this population allowed us to identify genes responsible for segregating traits as QTLs. Therefore, we considered that this RIL population was suitable for a comprehensive study of OPS in sorghum. Our genetic analysis identified a QTL on chromosome 5 that conferred leaf OPS, which corresponded to the *Organophosphate-Sensitive Reaction* (*OSR*).

## Results

### Assessing organophosphate pesticide sensitivity

**Figure 1:**
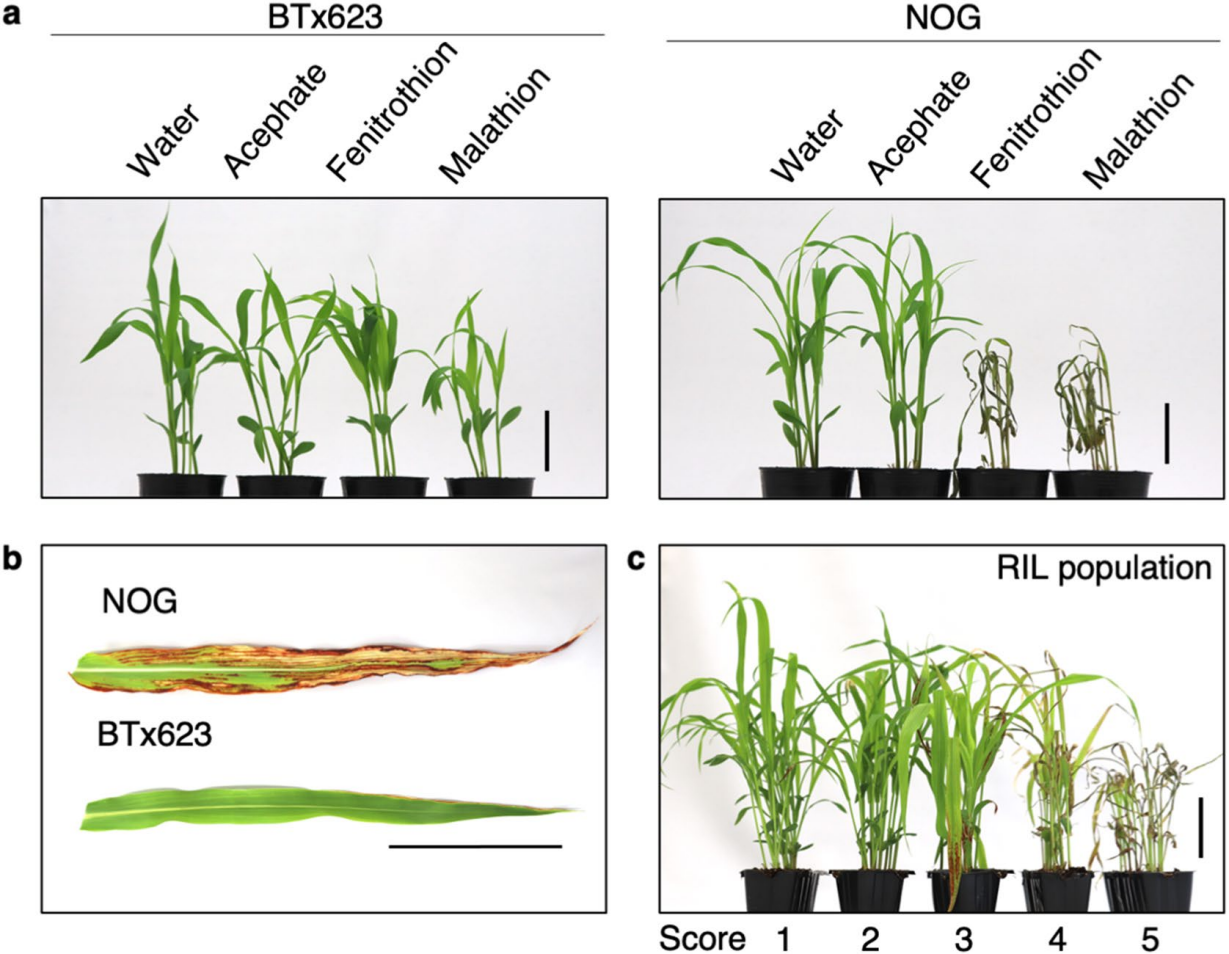
Phenotype of sorghum plants with organophosphate pesticide treatment. (**a**) Symptoms of BTx623 (left) and NOG (right) after acephate, fenitrothion, and malathion treatment. Scale bars; 5 cm. (**b**) OPS phenotypes of NOG and BTx623 in flag leaves, after spraying fenitrothion (1:500). Scale bars; 10 cm. (**c**) The phenotyping pool of OPS phenotypes segregating in the RIL population at the seedling stage. The mixture lines in each score with fenitrothion (1:500) spraying are displayed. Scale bars; 5 cm.

BTx623 and NOG displayed different responses to several types of organophosphate pesticides. Acephate (Ortran) was commonly used in our greenhouse to control aphids, and we noticed no visible growth defect throughout the entire growth period of both NOG and BTx623. In contrast, fenitrothion (Sumithion) and malathion (Marathion) caused severe necrosis only in NOG when applied to seedlings (Fig. 1a, chemicals structures of each pesticide are shown in Supplementary Fig. S1). This sensitivity was apparently holistic when fenitrothion was applied to field-growing NOG (Supplementary Fig. S2), excluding the possibility that OPS is confined developmentally. Necrotic lesions were also apparent when pesticides were spotted onto leaves at later growth stages (Fig. 1b). Indeed, we observed this OPS segregating in the RIL population, which prompted us to perform QTL analysis (Fig. 1c).

**Figure 2:**
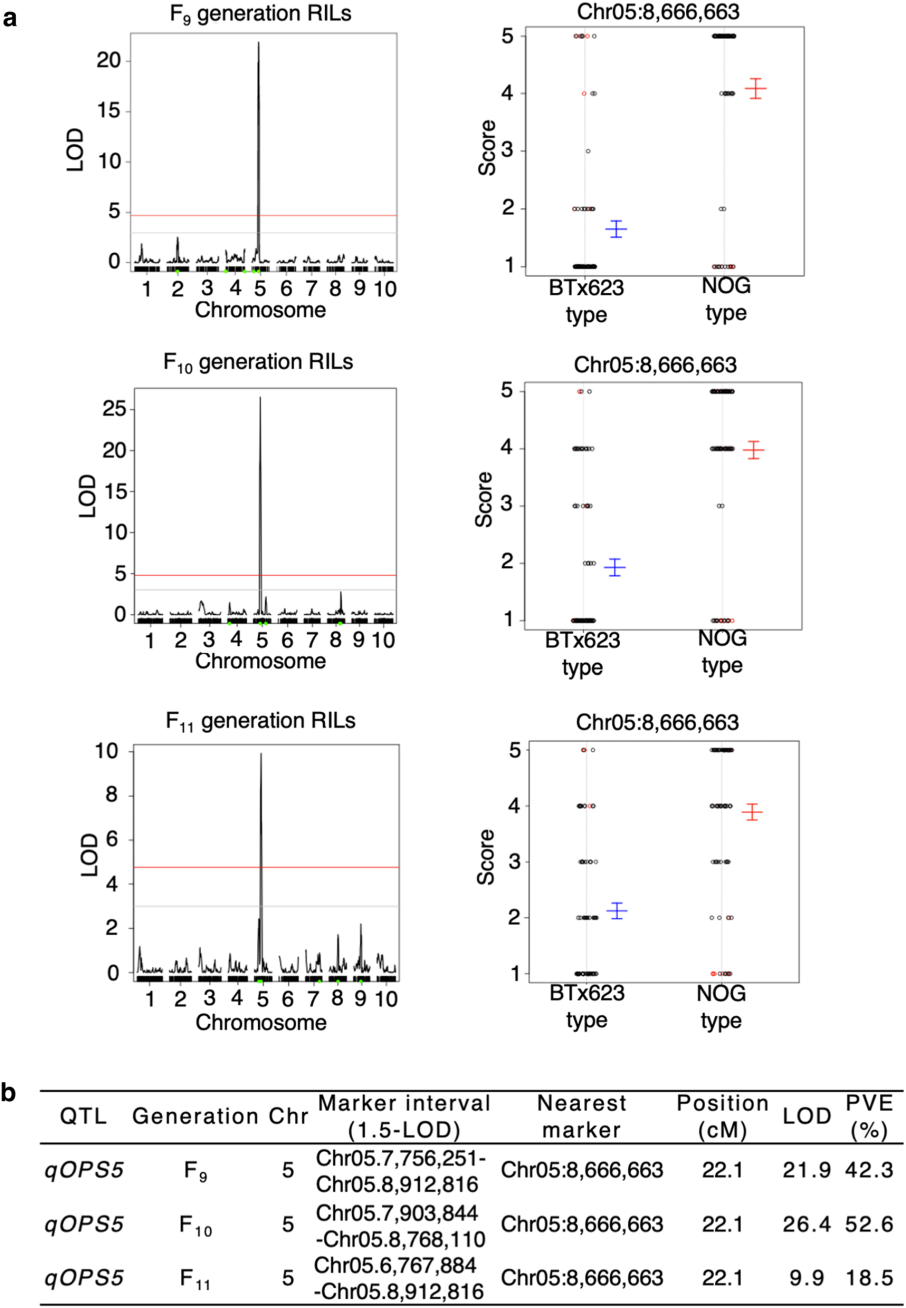
QTL analysis of OPS phenotype with fenitrothion treatment in the F_9_, F_10_, and F_11_ RIL populations. (**a**) The logarithm of odds (LOD) graphs for fenitrothion treatment using RILs in each population (left). The gray line represents an LOD threshold = 3, and the red line represents an LOD threshold based on a permutation test with 1000 iterations. Right panel represents plot phenotypes versus marker genotypes analysis corresponding to graphs. (**b**) Summary of QTLs identified in (**a**). The same QTL marker was obtained in the F_9_, F_10_, and F_11_ RIL generations. PVE indicates phenotype variation explained. Marker intervals were estimated based on confidence intervals (1.5-LOD).

QTL analysis of OPS using F_9_, F_10_, and F_11_ populations from the RIL showed a single peak near marker Chr05:8,666,663 on chromosome 5 after fenitrothion treatment (Fig. 2). An identical peak near marker chr05:8,666,663 was obtained on chromosome 5 with malathion treatment (Supplementary Fig. S3). The location of this QTL, termed *qOPS5* , on chromosome 5 was similar to those obtained in previous studies, including *organophosphate resistance* (*opr*)^42,43^, *resistance to chemical burning* (*rcb*)^44,45^, crop injury^24^, and *organophosphate-sensitive reaction* (*osr*)^46^, although the inbred lines used in each study differed. Given that no other prominent QTLs other than those on chromosome 5 were detected, we concluded that OPS in the RIL population is predominantly controlled by this single locus, *qOPS5*.

### Fine mapping

**Figure 3:**
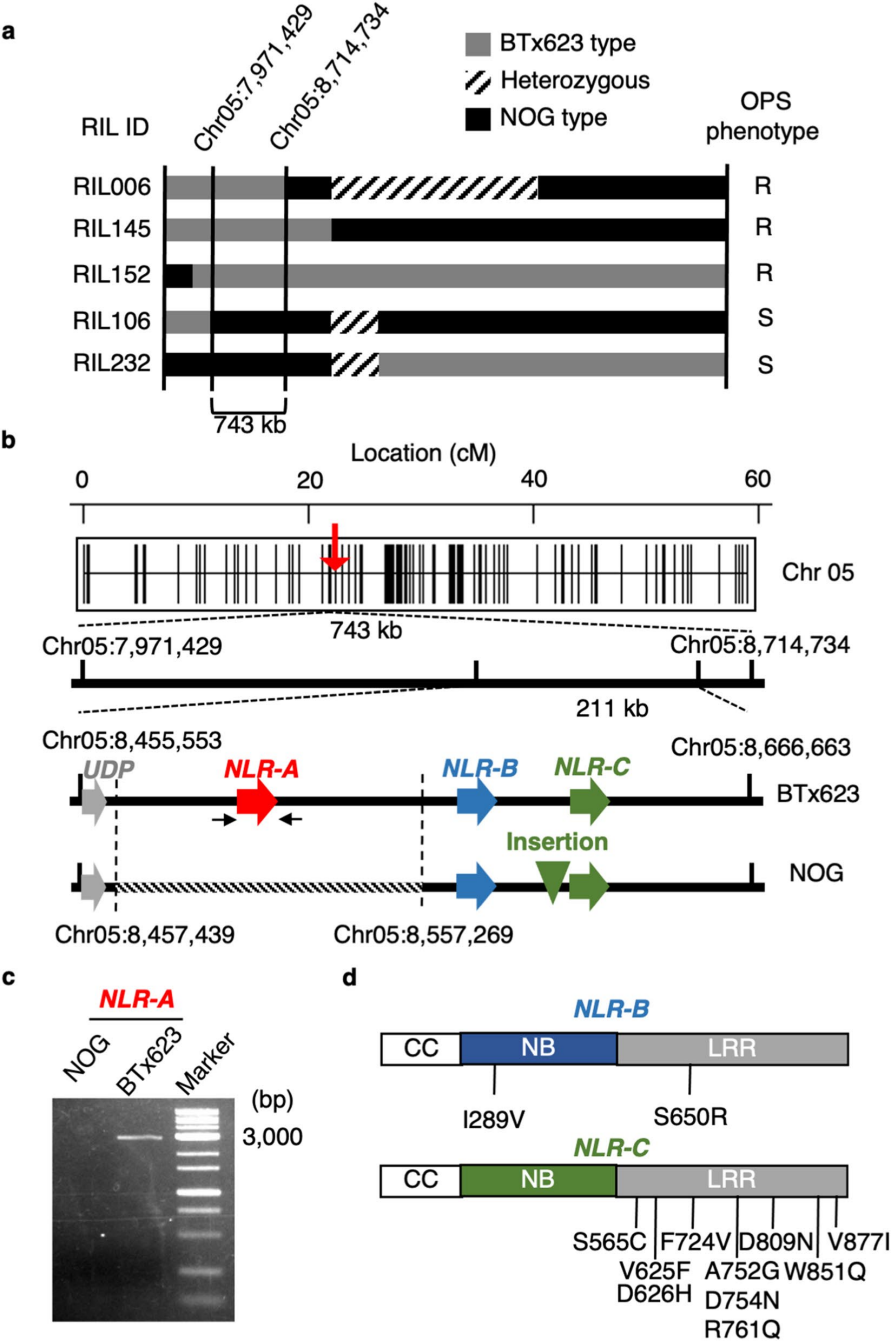
Three NB-LRR genes (*NLR-A*, *NLR-B*, and *NLR-C*) located in the *OSR* locus. (**a**) Graphical genotypes of RIL lines showing recombination at the putative *OSR* region on chromosome 5. *OSR* was mapped within a 743-kb region between SNP markers Chr05:7,971,429 and Chr05:8,714,734. The gray, striped, and black bars represent chromosomal regions homozygous for BTx623, heterozygous, and homozygous for NOG, respectively. ‘R’ and ‘S’ indicates lines resistant and sensitive to fenitrothion, respectively. (**b**) A closeup view of the putative *OSR* locus, located near SNP marker Chr05:8,666,663. Gray, red, blue, and green arrows indicate the positions of the UDP-glucoronosyl transferase gene, *NLR-A*, *NLR-B*, and *NLR-C*, respectively. The smaller black arrows indicate the relative location of primers used to amplify *NLR-A* in (**c**). The dashed line indicates the missing region from 8,457,439 to 8,557,269 including *NLR-A* in NOG. The green arrowhead indicates the 593-bp insertion upstream of *NLR-C* in NOG. (**c**) Agarose gel electrophoresis of PCR products corresponding to *NLR-A*, showing that NOG lacks this gene. The original image of the gel is provided in Supplementary Information 2. (**d**) Schematic diagrams showing the presumed coiled-coil (CC), nucleotide binding site (NB), and leucine-rich repeat (LRR) domains in NLR-B (upper) and NLR-C (lower) proteins. Predicted amino acid substitutions in NOG, compared with those in BTx623, are indicated.

The BTx623xNOG RIL population consists of 213 individuals, in which the genotyping of 3710 markers was determined in the F_6_ generation^39^. Displaying graphical genotypes of each RIL along with its OPS phenotype allowed us to narrow down the genomic region conferring OPS to a 743-kb region (Fig. 3a) between two SNP markers, Chr05:7,971,429 and Chr05:8,714,734. QTL analysis also showed that the QTL peaks were located at SNP marker Chr05: 8,666,663, encompassed by the chromosome regions detected from the recombination points (Fig. 3b). Within this region, there are 36 genes annotated in the database (https://phytozome.jgi.doe.gov, Phytozome version 12), in which a cluster of three NB-LRR genes (Sobic.005G071700, Sobic.005G071900, and Sobic.005G072000) existed in a tandem orientation (Fig. 3b, Supplementary Table S1). In fact, this cluster of three NB-LRR genes was reported as being likely to be responsible for leaf injury by Boyles et al. (2017) and for OPS of inbred line Nakei MS3B^46^. In this study, we termed it *OSR* in accordance with Kawahigashi et al. (2020) and further characterized these NB-LRR genes.

### Characterization of the *OSR* locus in NOG

We investigated the *OSR* locus in NOG, based on re-sequencing data of NOG we determined previously (DDBJ Sequence Read Archive Accession No. DRA008159). For simplicity, we termed three NB-LRR genes Sobic.005G071700, Sobic.005G071900, and Sobic.005G072000 as *NLR-A*, *NLR-B*, *and NLR-C*, respectively. First, comparison of these three genes with those in BTx623 showed that *NLR-A* was missing in NOG, lacking the corresponding genomic region from 8,457,439 to 8,557,269 on chromosome 5, which amounted to ~ 99.8 kb (Fig. 3b). Lack of *NLR-A* was subsequently confirmed by PCR specifically amplifying *NLR-A* (Fig. 3c). Given that OPS is dominant, involvement of *NLR-A* was unlikely (see below). Second, comparison of *NLR-B* and *NLR-C* indicated that both genes existed in NOG and BTx623. The *Sorghum bicolor* v3.1.1 database (https://phytozome.jgi.doe.gov, Phytozome version 12) predicted that *NLR-B* had no intron and encoded a 933 amino acid-long protein that had a nucleotide binding site domain (176–457 aa) and a leucine-rich repeat domain (483–903 aa). Similarly, *NLR-C* contained one exon of 2739 bp encoding 912 amino acids in both NOG and BTx623. We found that in NOG, *NLR-B* and *NLR-C* had two and ten amino-acid substitutions, respectively (Fig. 3d). For *NLR-B*, two amino-acid substitutions, I289V and S650R, were within the NB domain and LRR domain, respectively. The other polymorphism we detected in NOG was a 593-bp insertion, in the 5′ upstream region of *NLR-C* (Fig. 3b). Phylogenetic analysis of NLR-B and NLR-C proteins along with other NB-LRR proteins showed that NLR-B and NLR-C are structurally related to RPM1, which recognizes the AvrRpm1 type III effector avirulence protein from *P. syringae* in *Arabidopsis*^47^. They were also closely related to NB-LRRs in *Oryza sativa*, which leads to lesions on the leaf blade and broad-range resistance to the fungal pathogen *Pyricularia oryzae (syn. Magnaporthe oryzae)* and the bacterial pathogen *Xanthomonas oryzae pv. oryzae*, together with strong growth reduction (OsRLR1)^48^, and rice blast resistance (Pid3)^49^. (Supplementary Fig. S4).

### Segregation analysis

**Figure 4:**
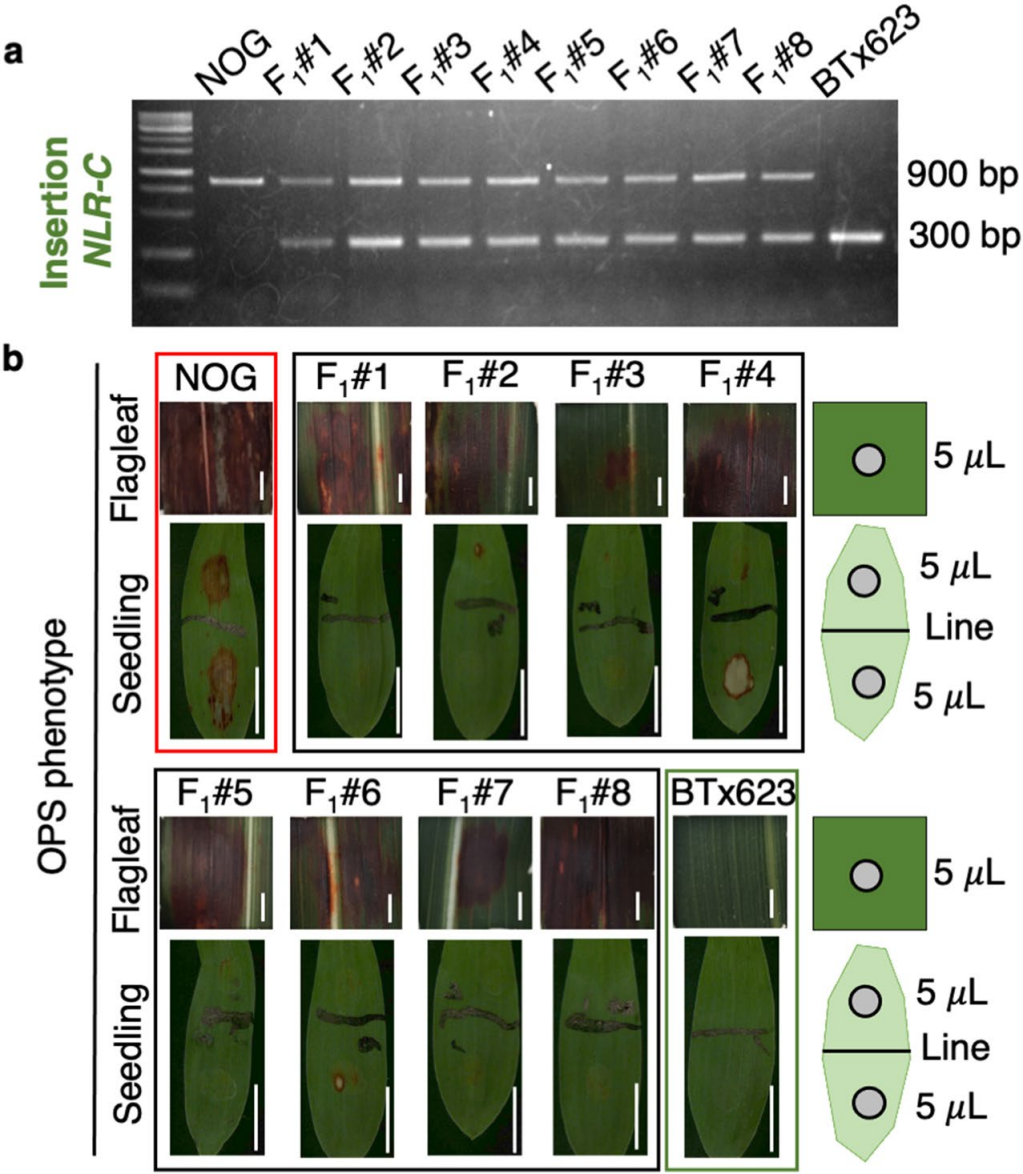
Semi-dominant OPS phenotype confirmed in F_1_ individuals. (**a**) Heterozygosity was confirmed by genotyping *NLR-C* with a pair of specific primers (NLR-C_Insertion_F and NLR-C_Insertion_R) that distinguish NOG-specific insertion (900 bp in NOG). The original image of the gel is provided in Supplementary Information 2. (**b**) F_1_ plants (#1–#8) were grown from seedling to mature stages along with NOG and BTx623, and fenitrothion was spotted onto the fourth or fifth leaves at the seedling stage as illustrated (lower panels. Scale bars; 1 cm) or sprayed onto mature leaves (upper panels, Scale bars; 0.6 cm).

We next tested whether *OSR* in NOG confers susceptibility in a dominant fashion as reported previously. Followed by confirmation of the F_1_ genotype by PCR, we investigated OPS in each F_1_ plant (Fig. 4). The results showed that the response to fenitrothion at the seedling stage was somewhat less severe and variable, whereas lesions were clearly observed on the mature leaves in all F_1_ plants. Although the precise reason for the unstable appearance of lesions at the seedling stage was unclear, it was apparent that all F_1_ plants exhibited OPS; based on these observations, we concluded that OPS is semi-dominant.

**Figure 5:**
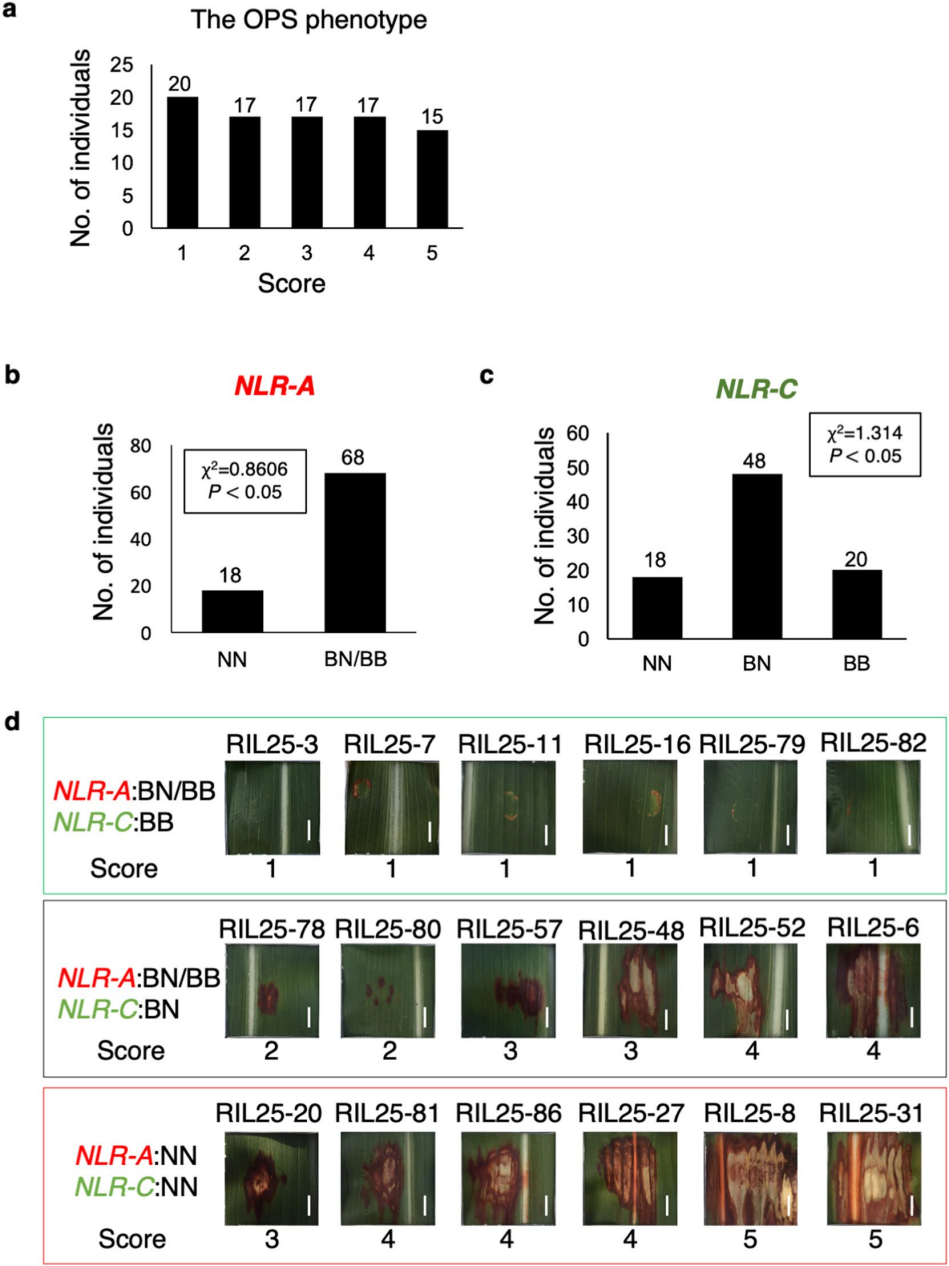
Linkage analysis of OPS with *NLR-C* in the RIL25-F_9_ segregating population. (**a**) Histogram of 86 individuals subjected to segregation analysis. The Y axis represents numbers of individuals and the X axis represents the scores, a score of 1 indicates resistance to OP treatment, whereas a score of 5 indicates sensitivity. (**b**) Segregation of *NLR-A* genotypes among F_9_ individuals showing a 1:3 ratio (*P* < 0.05, χ^2^ = 0.8606). (**c**) Segregation of *NLR-C* genotypes among F_9_ individuals showing a 1:2:1 ratio (*P* < 0.05, χ^2^ = 1.314). (**d**) Representative images of OPS phenotypes in the corresponding genotypes. The genotype of the *OSR* locus is indicated on the left, and the OPS phenotypes from individuals homozygous for BTx623 (top), heterozygous (middle), and homozygous for NOG (bottom) are shown. Scale bars; 0.6 cm.

To examine if the observed polymorphisms mentioned previously were indeed linked to the OPS phenotype, segregation analysis was performed. Our survey of RIL individuals showing intermediate OPS scorings in the F_8_ population found that RIL25 showed segregation of the *OSR* locus. Therefore, F_9_ progeny derived from an F_8_ plant heterozygous for the NB-LRR cluster was subjected to further segregation analysis. A total of 86 plants of RIL25 from the F_9_ generation were treated with fenitrothion at the heading stage in a greenhouse. Phenotyping of these plants indeed showed the segregation (Fig. 5a). Linkage analysis with genotyping data with *NLR-A* and *NLR-C* indicated that *OSR* segregated in a Mendelian fashion (Fig. 5b,c). Most importantly, co-segregation between OPS and NOG genotypes was found (Fig. 5d). These results strongly supported the notion that the *OSR* locus is semi-dominant and linked with *NLR-B* and *NLR-C*.

### Transcript accumulation of NB-LRR genes indicates altered expression in *NLR-C*

**Figure 6:**
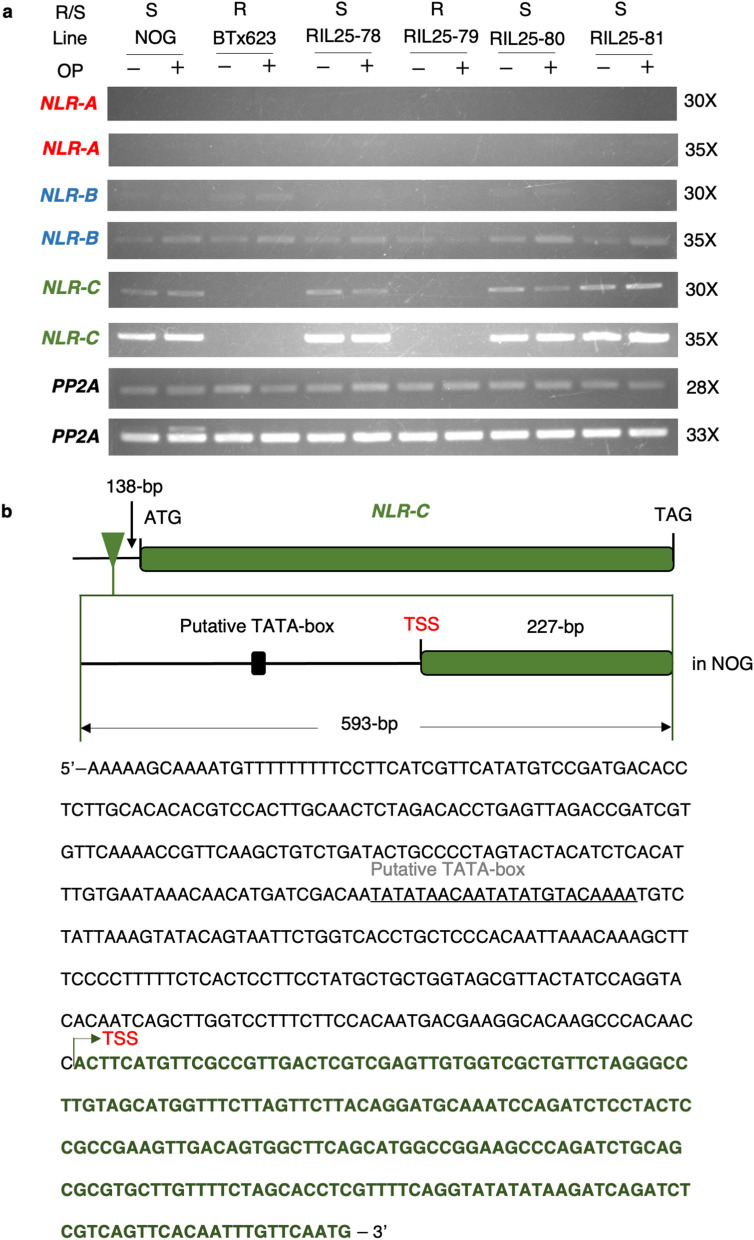
Expression of NLR genes in OSP and null mutation in *NLR-C*. (**a**) Transcript accumulation of the three NB-LRR genes (*NLR-A*, *NLR-B*, and *NLR-C*) estimated using semi-quantitative RT-PCR. Expression of *NLR-A*, *NLR-B*, and *NLR-C* in leaves. Accumulation of *NLR-C* transcripts coincides with the presence of the fenitrothion-sensitive (S) NOG genotype (NOG, RIL25-78, − 80, and − 81), whereas no transcript was detected in BTx623 and the resistant (R) line (RIL25-79). ‘+’ and ‘−’ indicate gene expression analysis in the presence or absence of organophosphate pesticide (OP), respectively. The *PP2A* gene was used as an internal control. The original gel images are provided in Supplementary Information 2. (**b**) Schematic diagram of the *NLR-C* coding region and its 5’ upstream region. *NLR-C* consists of a single exon (top), and the closeup view of the 593-bp insertion specific to NOG is shown below. Putative transcription start site (TSS, indicated by an arrow) is located 227 bp upstream from the 3′ end of the insertion. The nucleotide sequence of the insertion is shown below the diagram, where the putative TSS determined by 5′ RACE and the TATA-box sequence are indicated. A schematic diagram of the insertion region is shown, which contains the TSS and putative *TATA-box*, upstream *NLR-C* gene in NOG genetic background.

We performed semi-quantitative RT-PCR using gene-specific primers in the segregating RIL25 population along with the parental lines (Fig. 6a). RNA was extracted from mature leaves, either before or after fenitrothion treatment, and subjected to RT-PCR. For *NLR-A*, its transcript levels appeared to be extremely low and were not detectable in any samples irrespective of the presence (BTx623) or absence (NOG) of the gene. For *NLR-B*, we observed weak signals in all samples, and the expression level did not respond to fenitrothion treatment. In contrast, *NLR-C* was shown to be expressed at a substantial level and to be detectable by our RT-PCR analyses (30 and 35 cycles). Intriguingly, *NLR-C* was constitutively expressed in NOG regardless of fenitrothion treatment, whereas no signals corresponding to *NLR-C* transcripts were detected in BTx623. Consistent with this observation, RIL25 F_9_ segregants heterozygous for *OSR* and exhibiting OPS phenotype accumulated *NLR-C* mRNAs, whereas segregants homozygous for *OSR* and exhibiting resistance to fenitrothion was missing *NLR-C* transcripts (Fig. 6a).

### Functionality of *NLR-C* in NOG was accounted for by the presence of the promoter

To account for the differential expression of *NLR-C*, we reasoned that the 593-bp insertion present in NOG may positively affect transcription. This insertion is located 138 bp upstream of the ATG initiation codon of *NLR-C* extending towards the 5′ direction (Fig. 6b), suggesting that this insertion generates transcription initiation and/or part of 5′-untranslated region (UTR). Given that no transcript accumulated in BTx623, we postulated that the insertion contains a promoter activity and transcription initiates from this region. To examine this possibility, 5′-rapid amplification of cDNA ends (RACE) was performed with total RNA extracted from NOG and BTx623 with specific primers to detect the 5′ end of *NLR-C* transcripts (Supplementary Fig. S5a). The result showed that, as expected, the amplified band corresponding to the 5′ end was detectable only in NOG (Supplementary Fig. S5b). Sequencing of the amplified fragment demonstrated that the 5′ end lies in A at 365-bp upstream of ATG initiation codon (Supplementary Fig. S5c). Consequently *NLR-C* mRNA possesses a 365-bp 5′-UTR, within which no additional ORF was found. Furthermore, searches for core promoter elements in *O. sativa* in the database (http://bioinformatics.psb.ugent.be/webtools/plantcare/html) revealed that a putative promoter sequence, TATATAACAATATATGTACAAAA, exists 162-bp upstream of the start-site of *NLR-C* transcripts, which is also included in the 593-bp insertion (Fig. 6). Based on these results, we concluded that BTx623 has a null mutation in *NLR-C* due to the loss of a functional promoter, and that *NLR-C* is responsible for OPS in NOG.

## Discussion

Sensitivity to organophosphate pesticides has been reported in sorghum by several research groups, as *organophosphate insecticide reaction* (*opr*)^42,43^ and *resistance to chemical burning* (*rcb*)^45^. Although they mapped these loci using conventional DNA markers with different mapping populations, their location on chromosome 5 was suggested to represent the same locus^44^. Later, Boyles et al. (2017) characterized a ‘crop injury’ QTL as correlated with *opr*; the corresponding chromosomal region on chromosome 5 was shown to have an *NB-LRR* gene cluster, although no further experimental verification was provided^24^. More recently, Kawahigashi et al. (2020) investigated *organophosphate-sensitive reaction* (*osr*) using a sensitive inbred Nakei MS3B and raised the possibility that the same *NB-LRR* gene cluster is indeed responsible for OPS^46^. Based on the differential accumulation of transcripts in these three genes (termed *A*, *B*, and *C* in this study), *NLR-C* was supposed to be the major gene controlling OPS. The existence of amino-acid substitutions between Nakei MS3B and the resistant lines (BTx623 and Greenleaf) was also reported for *NLR-C*. Our current mapping of OPS observed in NOG was consistent with these previous reports. Detailed comparison of the nucleotide sequence along with transcript accumulation between BTx623 and NOG led us to conclude that *NLR-C* is the major gene.

Although these reports identified *NLR-C* as a candidate gene, a discrepancy exists over the dominance of OPS. In most of studies, OPS was shown to be dominant. However, only Kawahigashi et al. (2020) reported OPS in Nakei MS3B as recessive^46^. As for the expression of *NLR-C*, they showed that resistant BTx623 had no detectable transcripts, whereas sensitive Nakei MS3B had substantial levels of *NLR-C* transcripts, strongly arguing against the dominant role of resistance in BTx623. In fact, we demonstrated here that BTx623 has a null mutation due to the loss of promoter activity, which cannot cause a dominant effect on resistance. One possibility to explain this inconsistency may be the unstable appearance of OPS in the heterozygotes. In our study, a careful inspection of F_1_ plants showed that the lesion owing to OPS was weak and observed unstably at the seedling stage, whereas OPS became rather apparent in mature leaves (Fig. 4). These observations implied that the expression of OPS may be influenced developmentally, although the precise reason for this instability was unclear. We thus consider that re-evaluation is necessary to confirm whether OPS in Nakei MS3B is dominant.

Given the dominant effect of OPS in NOG, we further characterized the *NB-LRR* gene cluster. We excluded *NLR-A* because it is missing in NOG. Therefore, we considered that *NLR-B* and *NLR-C* are responsible for OPS. As for *NLR-B,* transcripts accumulated both in NOG and BTx623, whereas two amino acid substitutions in NOG (I289V and S650R) resides in the NB and LRR domains, respectively, and may account for the dominant effect of OPS. As for *NLR-C*, we have presented compelling evidence that the 593-bp deletion in BTx623 leads to the loss of promoter activity and a null mutation. Thus, a 593-bp insertion in the promoter region of *NLR-C* may be a determinant of the OPS phenotype in NOG. To test if *NLR-C* is involved and has dominant role in OPS, we performed heterologous complementation assays using agrobacterium infiltration in *Nicotiana benthamiana* (Supplementary Fig. S6). Overexpression of *NLR-C* and concomitant treatment with fenitrothion, however, did not result in necrotic lesions, as opposed to the control, suggesting that OPS is not simply conferred by NB-LRR and may require species-specific signaling cascades to respond to organophosphate pesticides. An alternative possibility is the requirement of both *NLR-B* and *NLR-C* to drive OPS.

OPS has been extensively characterized in tomato^25,27,50^. The *Fen* gene, which corresponds to the organophosphate pesticide fenitrothion sensitivity phenotypes, encodes a serine-threonine protein kinase^28^. The *Fen* and *Pto* genes share 87% amino acid similarity. Fen kinase is needed for fenthion sensitivity. Reports have shown that *Fen* stimulates organophosphate pesticide fenthion-inducible signaling and interacts with the N-terminal domain of a NB-LRR protein Prf^29^. Prf is indispensable for fenthion sensitivity, but the role of Fen and its association with Prf^51^ in fenthion sensitivity are poorly understood. Another example of an NB-LRR responding to chemicals is At5g46520 in *Arabidopsis*, which responds to accession-specific root growth arrest with DFPM^32,33^. DFPM was shown to be related to several abscisic acid (ABA) responses, including rapid disruption of ABA-induced stomatal closing and ABA activation of guard cell anion channels. Detailed analyses have uncovered that DFPM stimulates an ETI and results in rapid disruption of ABA signal transduction. A genetic variant of the NB-LRR gene in the *Arabidopsis* Columbia-0 accession is responsive specifically to DFPM by generating primary root meristem arrest.

Although there has been extensive research on R genes that defense against microbial pathogens, there are few studies on the toxic reaction in plants from organophosphate chemicals. In sorghum, the resistance to a necrotrophic fungus *Bipolaris sorghicola*, controlled by *ds1* encoding an NB-LRR has been reported^52^. Because *OSR* and *ds1* are distantly mapped on chromosome 5, the *OSR* locus is unlikely to be involved in the resistance to *B. sorghicola*^53^. Future study is required to test if any of the *NLR* genes in the *OSR* locus is associated with pathogens, similarly to *Pto/Fen* in tomato. NLR-C shows similarity to the NB-LRR protein Arabidopsis RPM1 (Supplementary Fig. S4). Notably, a mutation in one of the rice RPM1 homologs OsRLR1 resulting from ethyl methane sulfonate treatment led to spontaneous hypersensitive response-like lesions with strong growth reduction, which was similar to the OPS phenotype^48^. Based on the results in Supplementary Fig. S5, it appears that NLR-C indirectly recognizes fenitrothion. RPM1 indirectly recognizes the *Pseudomonas syringae* effectors AvrB and AvrRpm1 through an Arabidopsis RIN4 protein^54^. AvrB and AvrRpm1 phosphorylate RIN4, and RPM1 monitors this phosphorylation and causes ETI. It is possible that NLR-C employs a host protein(s) to sense fenitrothion. Alternatively, evidence has been accumulating that sensor and helper NB-LRR pairs work together to perceive pathogen effectors and induce ETI^55^. Conceptually, it is possible that NLR-B and NLR-C act in a complex with the organophosphate molecules or act downstream as part of the *OSR* signaling pathway.

Our previous analysis of QTLs related to biomass in the same population did not find a QTL on chromosome 5. Therefore, its impact on biomass is unlikely. However, the OPS phenotype might decrease production in sorghum upon pesticide application, and our understanding of the *OSR* locus presented in this study is important in future sorghum breeding. In particular, unstable appearance of OPS along with its dominance over resistance should be specially taken into consideration because it potentially affects F_1_ hybrids commonly used for commercial sorghum production. Our preliminary evaluation of OPS among the sorghum core collection used in our previous study indicated that most sorghum inbred lines are free from OPS. Nevertheless, two additional lines (IS11331 and Manfredi Cholila) were shown to have the same *OSR* haplotype as NOG and exhibit OPS at the seedling stage (Supplementary Fig. S7). Our previous admixture analysis categorized the inbred lines into three groups, where NOG belonged to Group II that represented Asian accessions^39^. While both NOG and Manfredi Cholila were clustered in Group II, IS11331 belonged to Group I. Although further study is required, these results may implicate that the rare OPS originate from multiple genetic backgrounds.

In conclusion, the presented results along with previous reports strongly suggest that the *OSR* locus corresponds to the *NB-LRR* gene cluster, particularly to *NLR-B* and *NLR-C. OSR* is dominant with the unstable appearance of OPS in the F_1_ generation, implicating particular caution in securing proper leaf longevity in breeding programs. Further investigations should allow us to understand the occurrence of OPS and its generic variation among sorghum cultivars, which may be related to diversification of the *NB-LRR* gene families.

## Methods

### Plant materials

Two parental lines, BTx623 and NOG, and the RIL population were established as described in our previous study^39^. All plant materials including each RIL generation were grown, harvested, and stored at the Institute of Plant Science and Resources (IPSR), according to the institutional guideline to handle plant materials. A genetic map was constructed using RAD-seq, and the genotyping data of 213 individuals were constructed in the F_6_ population. In this study, the F_9_, F_10_, and F_11_ populations were subjected to phenotyping of pesticide resistance, followed by QTL analysis. Phenotyping was carried out in multiple years with one replicate for each cultivation. Plants were germinated in small trays filled with vermiculite for approximately 10–14 days, subsequently transplanted to 30-cm diameter pots filled with soil from IPSR field. A single RIL line (RIL25) segregating for OPS was obtained by screening the F_8_ population. Seeds from RIL25 were harvested from an F_8_ individual that was heterozygous to *OSR* (confirmed by PCR), and the resulting F_9_ segregants were subjected to segregation analysis. F_1_ seeds were generated by crossing NOG with BTx623 with manual emasculation.

### Phenotyping OPS with organophosphate pesticide treatment

The trials were performed in an open-air greenhouse at the experimental field of IPSR (latitude: 34$^\circ$ 35′ 31″ N, longitude: 133$^\circ$ 46′ 7″ E), Kurashiki city, Okayama, Japan. Seedlings at the fourth-leaf stage were sprayed uniformly with 500 × diluted fenitrothion, malathion or acephate (Sumitomo Chemical Garden Products INC, Japan), and the phenotype was scored visually (from 1 to 5) at 7 days after the pesticide treatment. A score of 1 indicated essentially no leaf death, a score of 3 indicated approximately 50% of the leaf area was dead, and a score of 5 indicated 100% seedling (leaves and stem) death. The sensitive lines showed red-brown spots and necrotic lesions throughout their growth, and the resistant lines maintained greener leaf area. Flag leaves were treated with fenitrothion from F_1_ plants and the RIL25-F_9_ segregating population at the heading stage, and the greenness of plants was estimated visually 7 days after fenitrothion application. Moreover, evaluation of the greenness trait with fenitrothion treatment was performed for each RIL.

### QTL analysis

Genotype data obtained from RAD-seq in the F_6_ population was used to perform QTL analysis in this study. A total of 3710 SNPs distributed across the genome were obtained. A dense linkage map with 3710 markers spanning a distance of 644.8 cM on all chromosomes, with an average and maximum spacing between markers of 0.2 cM and 7.1 cM, respectively, was developed previously. Genotype probabilities were calculated using the calc.genoprobability function with a step size of 1 cM and an assumed genotyping error probability of 0.05 using the Kosambi map function as implemented in the R/qtl package^56,57^. QTL analysis was performed using the CIM function of the R/qtl package with the Haley–Knott regression method^58^. Linkage analysis was performed using the R/qtl package in R version 3.4.2. The LOD significance threshold for detecting QTLs was calculated by performing 1000 iterations using the R/qtl permutation test.

### Phylogenetic analysis of *OSR* genes

A phylogenetic tree using amino acid alignment of the three NB-LRR proteins (NLR-A, NLR-B, and NLR-C) and other typical R proteins from other species was constructed using a neighbor-joining tree^59^. The evolutionary distances were computed using the Poisson correction method^60^ and are in units of the number of amino acid substitutions per site. The R proteins used for phylogenetic analysis were as follows: *S. bicolor*, Sobic.005G071700 (NLR-A), Sobic.005G071900 (NLR-B), Sobic.005G072000 (NLR-C), Sobic.008G117000, Sobic.005G070500 and Sobic.001G291000. *O. sativa*, LOC_Os10g07978 (OsRLR1)^48^, LOC_Os06g22460 (Pid3)^49^, LOC_Os01g36640, LOC_Os10g07534 and LOC_Os04g46300. *A. thaliana*, AT3G07040 (RPM1)^47^. Phylogenetic analyses were performed using MEGA X^61^.

### Cloning of the *OSR* genes

The coding regions of the *NLR-B* and *NLR-C* genes were amplified by PCR using KOD FX Neo polymerase (TOYOBO, Japan) with specific primer pairs, InFusion_NLR-B_Xho1_fw and InFusion_NLR-B_Not1_rv, InFusion_NLR-C_EcoR1_fw and InFusion_NLR-C_BamH1_rv, respectively. The program involved: an initial denaturation step at 94 °C for 2 min, 35 cycles of denaturation step at 98 °C for 10 s, annealing step at 60 °C for 30 s and extension step at 72 °C or 5 min, and a final extension at 72 °C for 10 min. The PCR fragment of *NLR-B* was cloned using an In-Fusion HD cloning kit (Takara, Japan), into pGreen0029_35S digested and linearized with *Xho*1 and *Not*1. Likewise, the PCR fragment of *NLR-C* was cloned into pGreen0029_35S digested with the *Eco*R1 and *Bam*H1. Deletion of *NLR-A* in NOG was examined by PCR amplification of genomic DNA templates using KOD FX Neo polymerase (TOYOBO, Japan).

### Gene expression analysis

Flag leaves at the heading stage were used for expression analysis of *NLR-A*, *NLR-B*, and *NLR-C*. Total RNA was extracted from plant samples using an RNeasy Plant Mini Kit (Qiagen, Germany). First-strand cDNA was synthesized from 500 ng of each RNA sample in a 50 µL reaction solution using the SuperScript III First-Strand System (Thermo Fisher Scientific, USA). PCR was performed using 1 µL aliquots of cDNA solution in a 25-µL volume with KOD FX Neo DNA polymerase (TOYOBO, Japan). The PCR profile was as follows: initial denaturation for 2 min at 94 °C 28–35 cycles of 15 s at 94 °C 30 s at 60 °C and 1 min at 68 °C then 5 min at 68 °C or final extension. Five microliter aliquots of the PCR products were analyzed by electrophoresis in 1% agarose gels. Primers used in this study are listed in Supplementary Table S2.

### 5′-RACE analysis of candidate gene

The 5′ RACE experiment was performed using a 5-Full RACE Core Set Kit (TAKARA, Japan) in accordance with the manufacturer’s instructions. Fifteen microliters of ligated cDNA was then used as a template for nested PCR using KOD FX Neo polymerase (TOYOBO, Japan) with two sets of primers, the outside set was sense primer_1 and antisense primer_1, and the inside set is sense primer_2 and antisense primer_2. The PCR reaction mixture was incubated for 2 min at 94 °C followed by 30 amplification cycles, comprising denaturation at 94 °C for 30 s, annealing at 60 °C for 30 s and extension at 68 °C for 30 s. The reaction was extended for another 10 min at 68 °C to ensure full extension. Primers used in this study are listed in Supplementary Table S2.

### Transient expression of *NLR-C* in* Nicotiana benthamiana*

Agroinfiltration of *N.* *benthamiana* was performed as described previously^62^. *A.* *tumefaciens* strain GV3101, carrying *NLR-C*, Pit D485V, or GFP was used to infiltrate leaves of 5-week-old *N*. *benthamiana* plants. p19 silencing suppressor was used to enhance gene expression. Each agrobacterium culture was resuspended in buffer containing 10 mM MgCl_2_, 10 mM MES, pH 5.6, and 150 µM acetosyringone, and incubated at room temperature for 2–3 h before infiltration. After incubation for 24 h, fenitrothion (1:7500 dilution) or distilled water were infiltrated into the same spots as used for agroinfiltration. The photosynthetic capacity was measured at 3 days post-inoculation (dpi) using an imaging fluorimeter (FluorCam 800MF, photon systems instruments). Infiltrated plants were kept at 22 °C. Photographs were taken at 17 days post-inoculation (dpi) for the assessment of cell death.

## Supplementary Information

Supplementary Information 1.

Supplementary Information 2.
