# Supplementary material for: NB-LRR-encoding genes conferring susceptibility to organophosphate pesticides in sorghum
Source: Sci Rep. 2021 Oct 6;11:19828. doi: 10.1038/s41598-021-98908-7 (PMC8494876; doi:10.1038/s41598-021-98908-7)
Supplement: Supplementary file 1 — Supplementary Information 1. [file 41598_2021_98908_MOESM1_ESM.pdf]

Supplementary Information 1

NB-LRR-encoding genes conferring susceptibility to organophosphate pesticides in sorghum

Zihuan Jing[1], Fiona Wacera W.[1], Tsuneaki Takami[1], Hideki Takanashi[2], Fumi Fukada[1], Yoji Kawano[1], Hiromi Kajiya-Kanegae[3], Hiroyoshi Iwata[2], Nobuhiro Tsutsumi[2], and Wataru Sakamoto[1]*

[1]Institute of Plant Science and Resources, Okayama University, Kurashiki, Okayama 710-0046, Japan

[2]Graduate School of Agricultural and Life Sciences, The University of Tokyo, 1-1-1 Yayoi, Bunkyo-ku, Tokyo 113-8657, Japan

[3]Research Center for Agricultural Information Technology, National Agriculture and Food Research Organization, Minato-ku, Tokyo 105-0003, Japan

*Corresponding Author

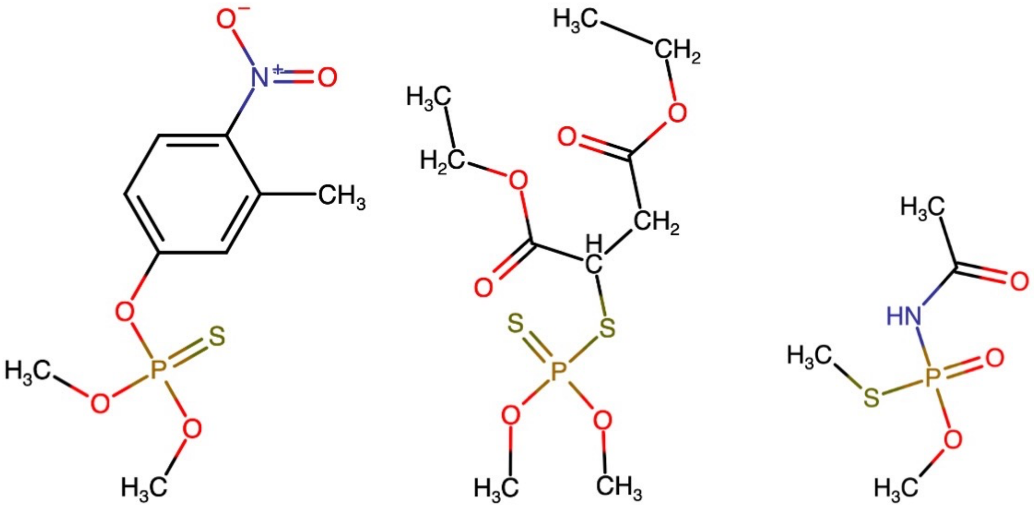

Fenitrothion (Sumithion) | Malathion (Marathion) | Acephate (Ortran)

Supplementary Figure S1 Chemical structure of fenitrothion (Sumithion), malathion (Marathion) and acephate (Ortran) used in this study.

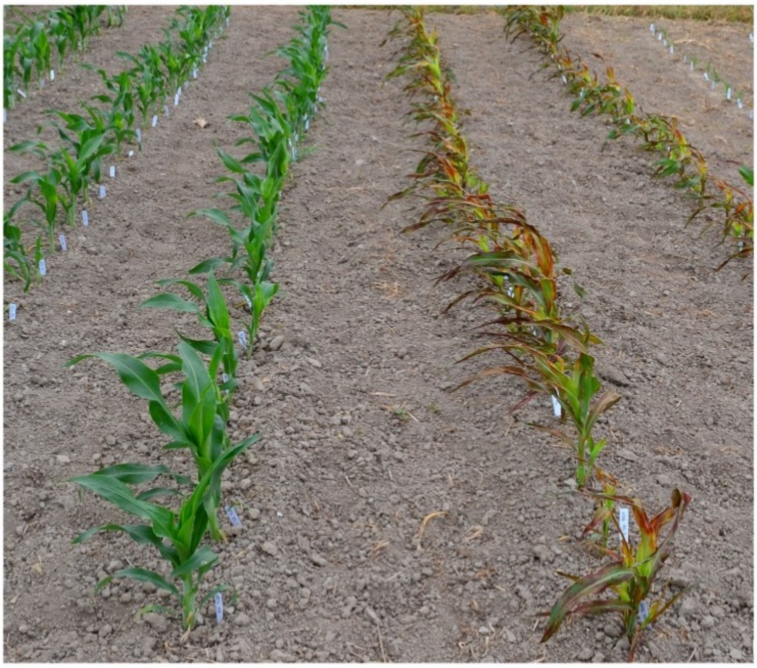

Supplementary Figure S2 Example of organophosphate sensitivity observed in the field-grown plants. Plants grown for approximately three weeks after transplantation were sprayed with 1,000X diluted Fenitrothion. The photograph was taken two days after the treatment (Left: Btx623, Right: NOG).

a

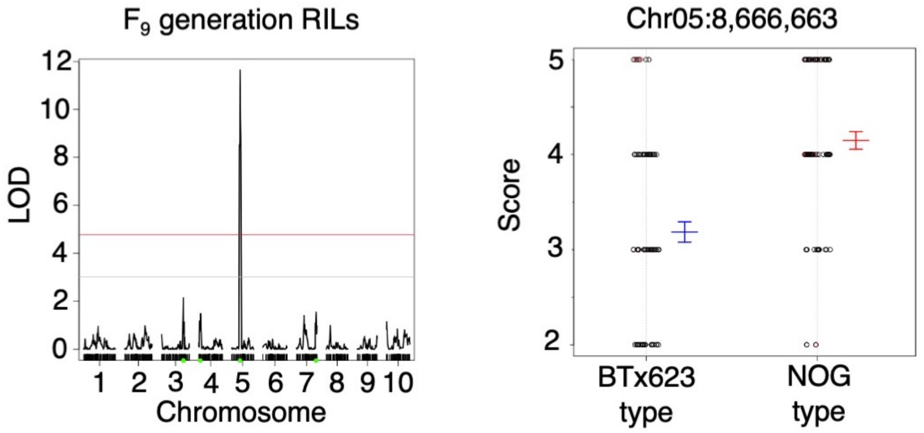

b

QTL	Treatment	Generation	Chr	Marker interval	Nearest marker	Position (cM)	LOD	PVE (%)
qOPR5	Malathion	F_9	5	Chr05.7,903,844 -Chr05.8,912,816	Chr05:8,666,663	22.1	11.6	22.7

Supplementary Figure S3 QTL analysis of the OPS phenotype with malathion treatment resistance in the F_9 RIL population.

(a) The left panel shows the logarithm of odds (LOD) graph for malathion treatment in the RILs. A single peak was found near marker Chr05:8,666,663 responding to malathion treatment. The gray line represents an LOD threshold = 3, and the red line represents an LOD threshold based on a permutation test with 1000 iterations. The right panel represents plot phenotypes versus marker genotypes corresponding to the graphs, with the mean of BTx623 type (blue) and NOG type (red).

(b) Summary of the QTL identified for malathion treatment. PVE is the percentage phenotypic variation explained by a QTL. Marker intervals were estimated based on confidence intervals (1.5-LOD).

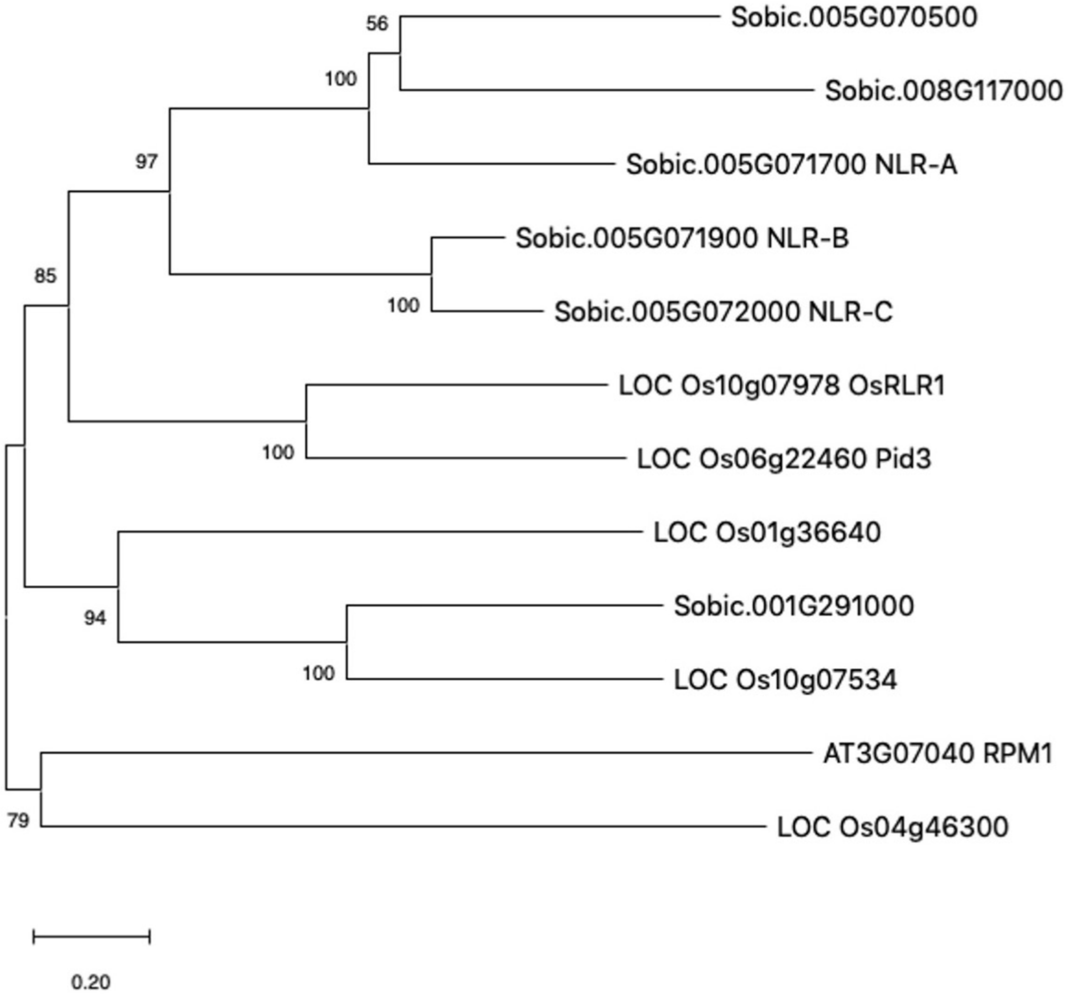

Supplementary Figure S4 A phylogenetic tree of NLR-A, NLR-B, and NLR-C with other NB-LRR proteins. The protein sequences were aligned using MEGAX, and the results are displayed graphically using a Neighbor-Joining plot. Bootstrap values from 1,000 bootstrap replicates are shown at the nodes for assessment of the robustness of the tree.

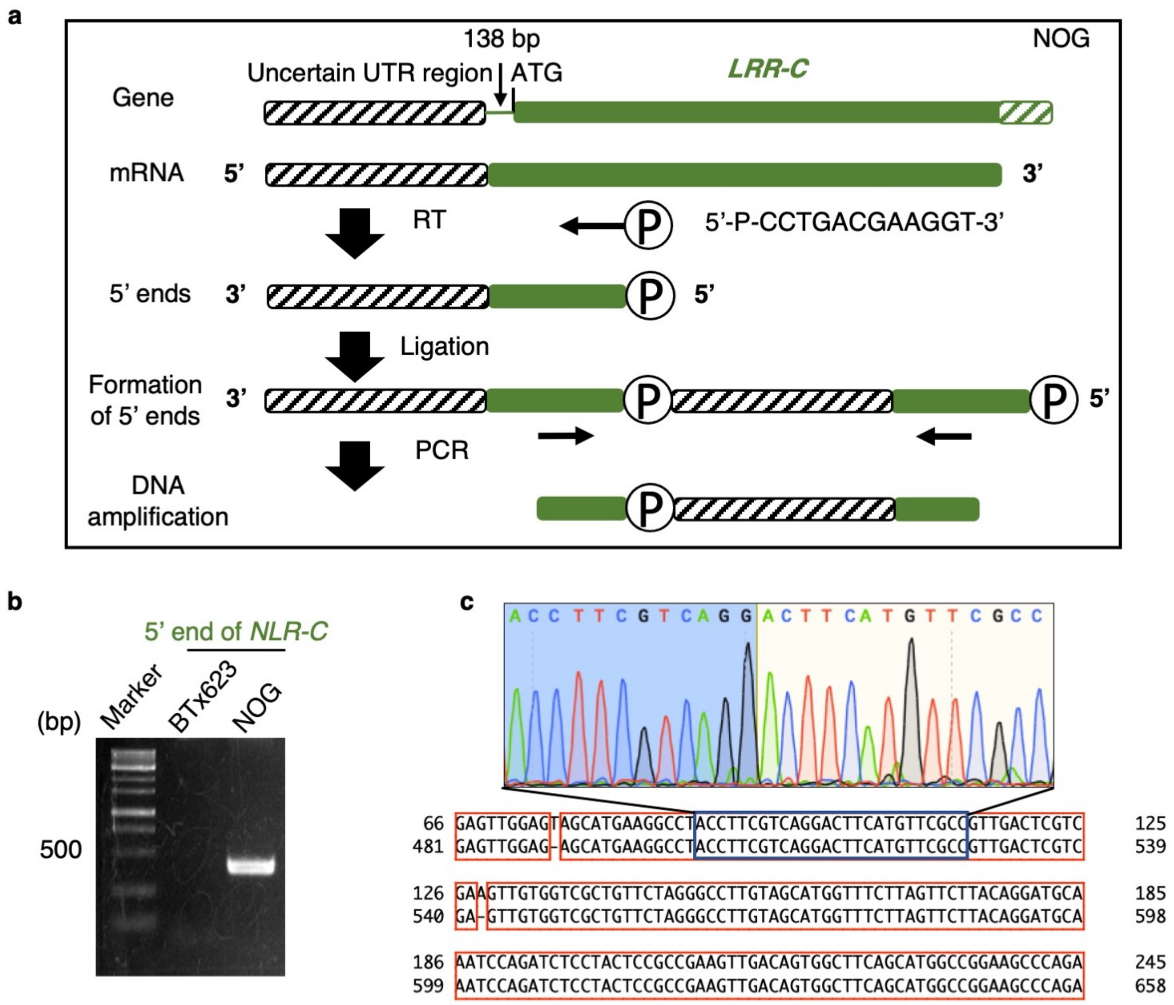

Supplementary Figure S5 Detection and determination of the 5' end of *NLR-C* transcripts by 5' RACE.

(a) Schematic diagram of 5' RACE workflow using a Takara 5'-Full RACE Core Set. Black striped bars indicate the unknown 5' UTR region to be determined: a green thin line indicates 138 bp of known sequence from the ATG code in BTx623: green thick bars indicate the coding sequence of *NLR-C*. A horizontal black arrow with letter P represents the 5' end-phosphorylated RT primer, and two horizontal black arrows represent the pair of nested primers to amplify the PCR fragment that contains the 5' UTR region.

(b) Agarose gel electrophoresis of the PCR fragment from 5' RACE, showing that the fragment was detectable only in NOG. The original gel image is provided in Supplementary Information 2.

(c) Nucleotide sequence chromatograms showing the junction between the RT primer (blue background) and the 5' UTR (cream background) containing the transcription start site. Corresponding sequences are indicated at the bottom.

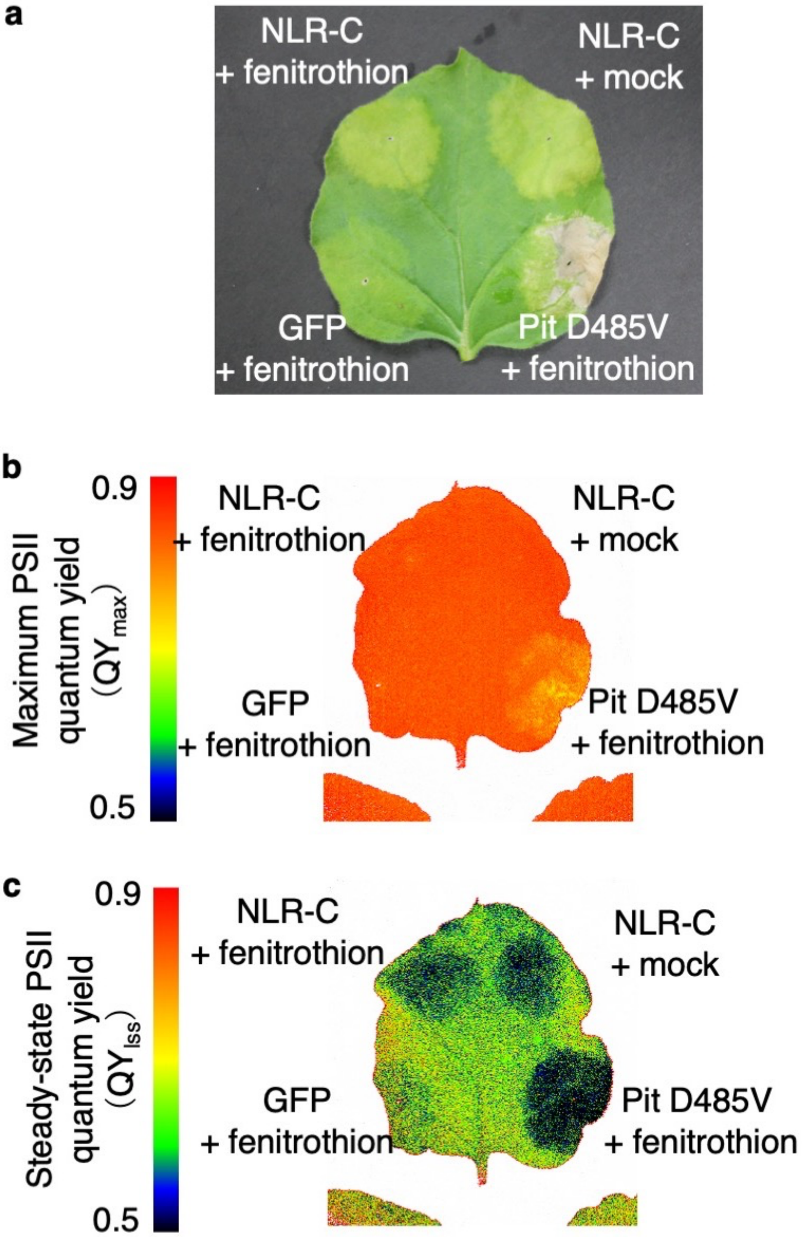

Supplementary Figure S6 Transient expression of *NLR-C* in *N. benthamiana*. *A. tumefaciens* strain GV3101, carrying *NLR-C*, Pit D485V, and GFP was used to infiltrate the leaves of 5-week-old *N. benthamiana* plants. Pit D485V is a constitutively active mutant of a rice NB-LRR Pit and this mutant autonomously induces cell death. The experiment was done three times independently with similar results.

(a) Photographs were taken at 17 days post-inoculation (dpi) for cell death.

(b) Maximum PSII quantum yield (Fv/Fm) measured at 3 days post-inoculation (dpi)

(c) Steady-state PSII quantum yield at 3 dpi.

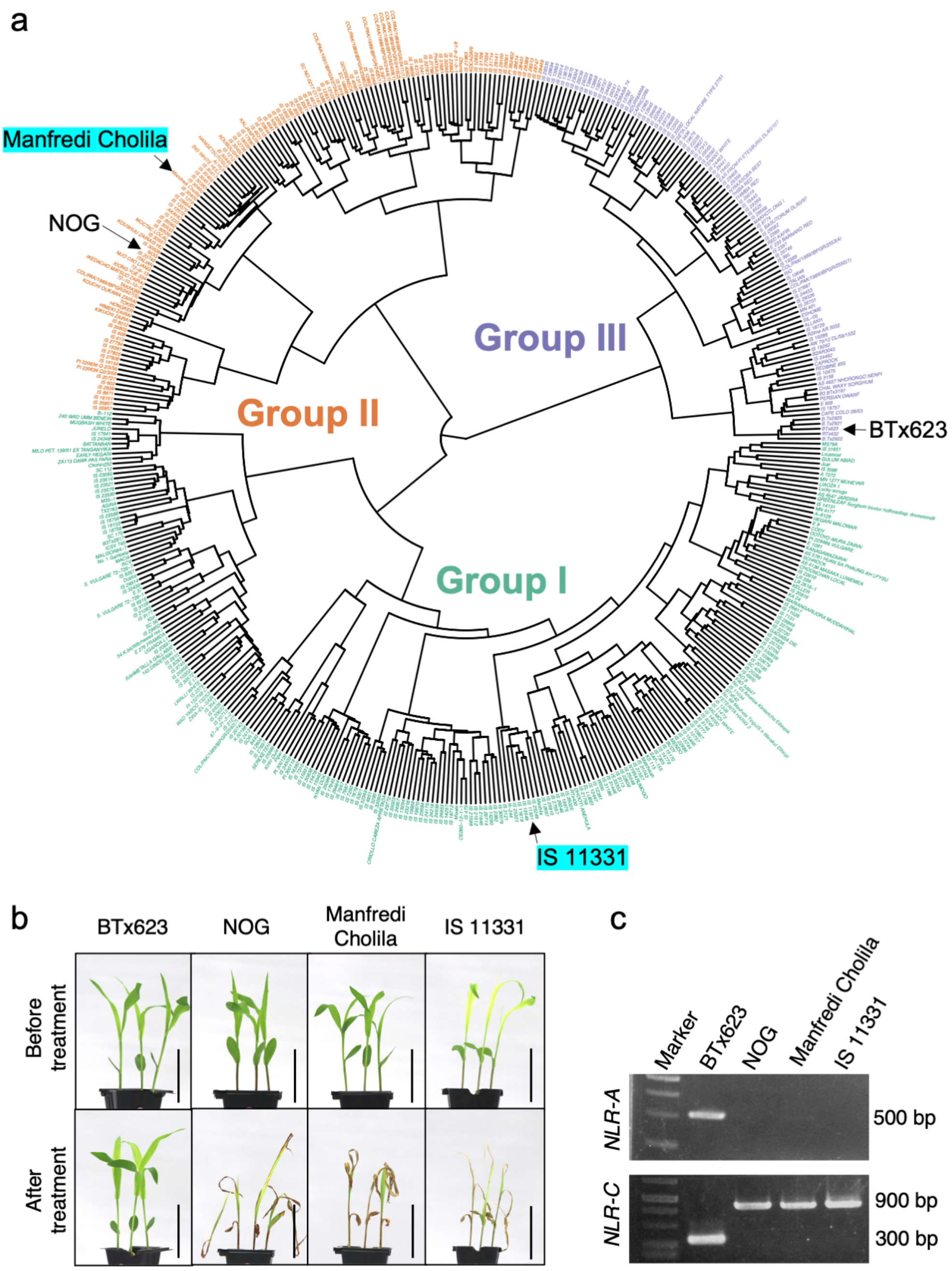

Supplementary Figure S7 Two additional lines showing OPS similarly to NOG.

(a) Ward's hierarchal clustering of the accessions from our previous work (Kajiya-Kanegae et al., 2020). Positions of NOG and BTx623 are indicated by the arrows, along with two additional accessions (Manfredi Cholila and IS11331) showing sensitivity to Fenitrothion (highlighted in blue).

(b) Representative images of OPS phenotypes 4 days after Fenitrothion treatment. Scale bar= 5 cm.

(c) Haplotype of Manfredi Cholila and IS11331 assessed by *NLR-A* and *NLR-C* genotypes. The original gel images are provided in Supplementary Information 2.

SupplementaryTable S1 Fine mapping of *qOPS5*. A total of 36 genes were found within a 743-kb candidate region, and the root/shoot expression level in BTx623

ID	No.	Position		Holomogs	Description of best ortholog(s)	FPKM	
						root	shoot
Sobic.005G068900	1	7983818-7985664	Calcium-binding EGF domain (EGF_CA) // Protein tyrosine kinase (Pkinase_Tyr)	LOC_Os09g38910	OsWAK92 - OsWAK receptor-like protein kinase, expressed	2.5	none
Sobic.005G069000	2	8002552-8003422	Wall-associated receptor kinase galacturonan-binding (GUB_WAK_bind)	none		2.8	none
Sobic.005G069166	3	8033568-8037796	Protein kinase domain (Pkinase) // Wall-associated receptor kinase galacturonan-binding (GUB_WAK_bind)	LOC_Os09g38850	OsWAK91 - OsWAK receptor-like protein kinase, expressed	none	none
Sobic.005G069332	4	8039629-8040667	Wall-associated receptor kinase galacturonan-binding (GUB_WAK_bind)	none		none	none
Sobic.005G069500	5	8059982-8068643	Calcium-binding EGF domain (EGF_CA) // Protein tyrosine kinase (Pkinase_Tyr) // Wall-associated receptor kinase galacturonan-binding	LOC_Os09g38910	OsWAK92 - OsWAK receptor-like protein kinase, expressed	3.8	none
Sobic.005G069700	6	8083872-8085723	Calcium-binding EGF domain (EGF_CA) // Protein tyrosine kinase (Pkinase_Tyr)	LOC_Os02g56370	OsWAK20 - OsWAK receptor-like protein kinase, expressed	7	none
Sobic.005G069800	7	8086049-8091371	Calcium-binding EGF domain (EGF_CA) // Wall-associated receptor kinase galacturonan-binding (GUB_WAK_bind)	none		3.4	none
Sobic.005G069900	8	8111386-8120604	Wall-associated receptor kinase galacturonan-binding (GUB_WAK_bind)	none		none	none

Sobic.005G070000	9	8121398-8124011	Calcium-binding EGF domain (EGF_CA) // Protein tyrosine kinase (Pkinase_Tyr)	LOC_Os02g56370	OsWAK20 - OsWAK receptor-like protein kinase, expressed	0.5	none
Sobic.005G070101	10	8151228-8152810	no annotation	none		none	none
Sobic.005G070200	11	8205513-8212500	Calcium-binding EGF domain (EGF_CA) // Protein tyrosine kinase (Pkinase_Tyr) // Wall-associated receptor kinase galacturonan-binding	LOC_Os09g38910	OsWAK92 - OsWAK receptor-like protein kinase, expressed	2.5	none
Sobic.005G070301	12	8216267-8225543	similar to DNA-directed RNA polymerases I, II, and III subunit RPABC5	none		none	none
Sobic.005G070400	13	8221154-8225543	similar to At5g63940 (kinase with adenine nucleotide alpha hydrolases-like domain-containing protein)	0At5g63940		29.6	5.6
Sobic.005G070500	14	8284386-8287550	LEUCINE-RICH REPEAT-CONTAINING PROTEIN	ChrUn.fgenesh.gene.29	expressed protein	none	none
Sobic.005G070600	15	8299163-8299673	no annotation	none		none	none
Sobic.005G070700	16	8303332-8303938	no annotation	none		none	none
Sobic.005G070800	17	8312771-8313340	no annotation	none		none	none
Sobic.005G070850	18	8316735-8317291	no annotation	none		none	none
Sobic.005G070901	19	8328453-8329298	no annotation	none		none	none

Sobic.005G070951	20	8330915-8332367	Domain of unknown function (DUF1719)	LOC_Os04g01560	expressed protein	2	0.1
Sobic.005G071000	21	8343530-8344320	zinc-binding in reverse transcriptase (zf-RVT)	none		0.2	0.1
Sobic.005G071100	22	8367040-8370433	proliferating cell nuclear antigen (PCNA)	none		none	none
Sobic.005G071200	23	8380598-8381133	no annotation	none		none	none
Sobic.005G071250	24	8389620-8390266	no annotation	none		none	none
Sobic.005G071300	25	8427296-8431495	weakly similar to Os10g0331600 protein	none		4.5	0.5
Sobic.005G071401	26	8438160-8439218	no annotation	none		none	none
Sobic.005G071500	27	8455553-8457370	UDP-GLYCOSYLTRANSFERASE 83A1	LOC_Os10g18510		0.1	none
Sobic.005G071700	28	8492625-8496305	similar to LRR19	none		1.5	none
Sobic.005G071800	29	8552940-8555677	no annotation	none		4	2.2
Sobic.005G071900	30	8560707-8564559	similar to LRR19	none		26	none
Sobic.005G072000	31	8587677-8590415	LEUCINE-RICH REPEAT-CONTAINING PROTEIN	ChrUn.fgenesh.gene.29	expressed protein	0.7	none
Sobic.005G072100	32	8593373-8604479	similar to Preprotein translocase secA subunit	none		5.4	9

Sobic.005G072200	33	8664614-8668855	similar to Lipase, putative	none		15	25
Sobic.005G072300	34	8695058-8697289	similar to Transmembrane amino acid transporter protein	none		none	3.7
Sobic.005G072400	35	8699165-8701533	PTHR10209:SF170 - 2-OXOGLUTARATE (2OG) AND FE(II)-DEPENDENT OXYGENASE SUPERFAMILY PROTEIN	LOC_Os10g14180	expressed protein	1.2	1.8
Sobic.005G072500	36	8723163-8725266	similar to Transmembrane amino acid transporter protein	none		none	17

FPKM: fragments per kilobase of exon per million reads mapped. The raw data come from Sorghum Functional Genomics Database (http://structuralbiology.cau.edu.cn/sorghum).

Supplementary Table S2 Oligonucleotides used as primers in this study

Primer name	Nucleotide sequence (5'-sequence-3')
RT-P5end*	CCTGACGAAGGT
Sense primer_1	TGCTGATTGGCAAGCTAGGT
Antisense primer_1	GCCGTGACCTCCAAACCTAA
Sense primer_2	TTGGCGAAAGAAGCAGCAAC
Antisense primer_2	ACCAGTCCAAGTATCAGAGGT
*NLR-A*_F	TGACGAGTTCATGTACAAGCTTCATGGCA
*NLR-A*_R	CTCATGTTCATAGTGCTAGAATTGACTGGC
*NLR-B*_F	ATTGTCTTTCAAACAACCAACACATT
*NLR-B*_R	TTCGGCAACACTGCACTCGTTCAGCTG
*NLR-C*_F	CCGTGCAGTCTATGTTTCTACACA
*NLR-C*_R	CCTAGGCACCTTTACTCCACGCTG
*NLR-C*_Insertion_F	ACGCGTGAGGGCGGATGTAAGGTT
*NLR-C*_Insertion_R	AGTGCTCAGAGTCACTAGTGTACCCG
*NLR-A*_CDS_F	CTCAGCGCATGCAACACAACCACC
*NLR-A*_CDS_R	ATCCAGTAGCTAGCATCAAAGCCT
InFusion_NLR-B_XhoI_fw	CGGGCCCCCCCTCGAGATGGCAGAGGCTGTTGTGG
InFusion_NLR-B_NotI_rv	ACCGCGGTGGCGGCCGCTCAGCCAGCGAA
InFusion_NLR-C_EcoRI_fw	GCTTGATATCGAATTCATGGCGGAGGCCGTCGTG
InFusion_NLR-C_BamHI_rv	TAGAACTAGTGGATCCCTAATTAAAAAAA

* 5' end was phosphorylated.
