# Supplementary material for: NB-LRR-encoding genes conferring susceptibility to organophosphate pesticides in sorghum
Source: Sci Rep. 2021 Oct 6;11:19828. doi: 10.1038/s41598-021-98908-7 (PMC8494876; doi:10.1038/s41598-021-98908-7)

Supplementary Information 2

Original gel images

Figure 3c

Figure 4a

Figure 6a

Figure S5b

Figure S7c

Figure 3c

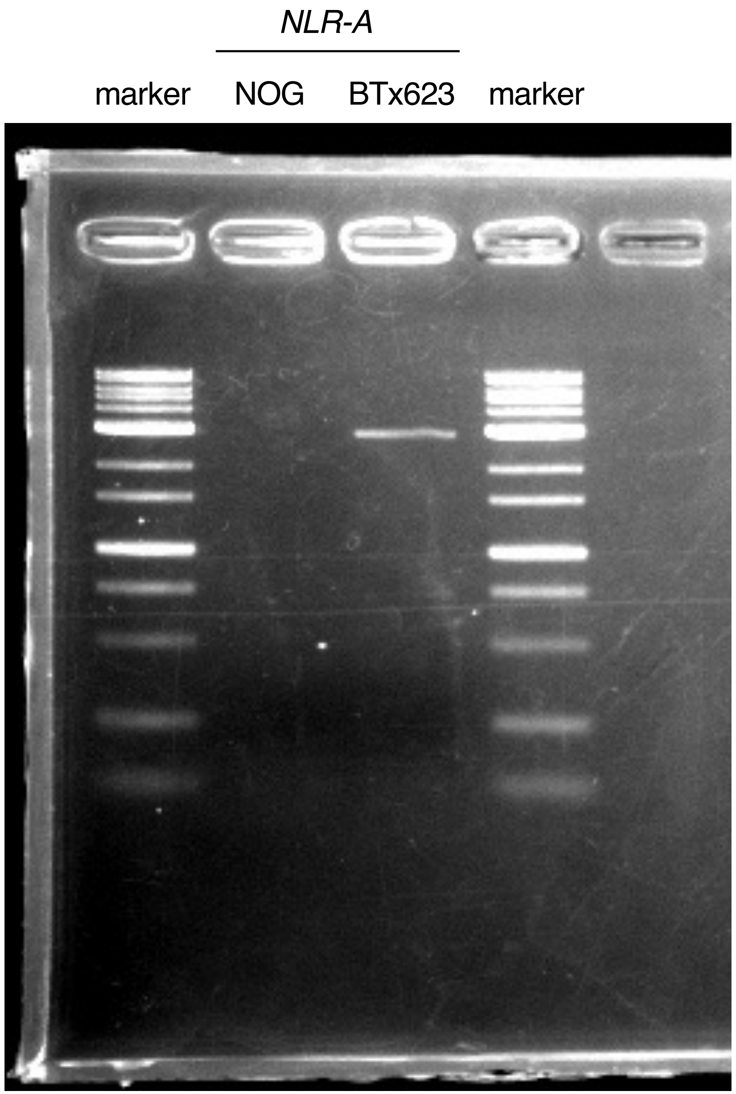

Figure 4a

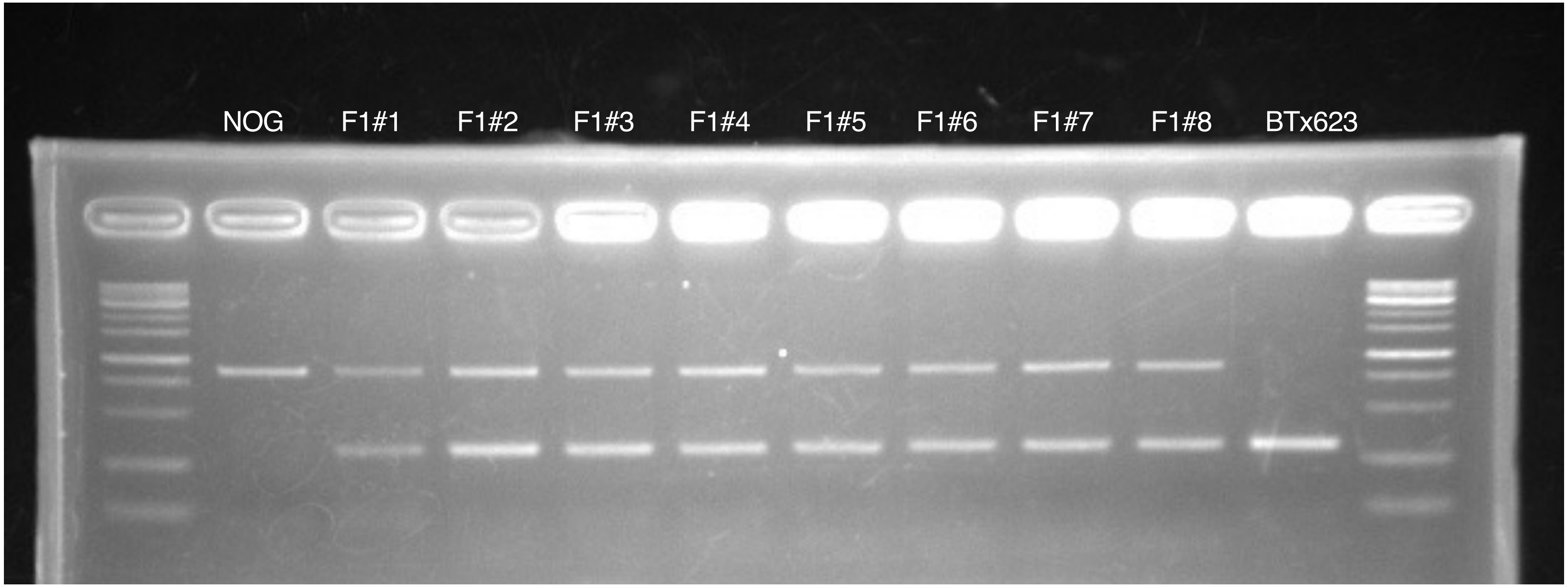

Figure 6a

PP2A

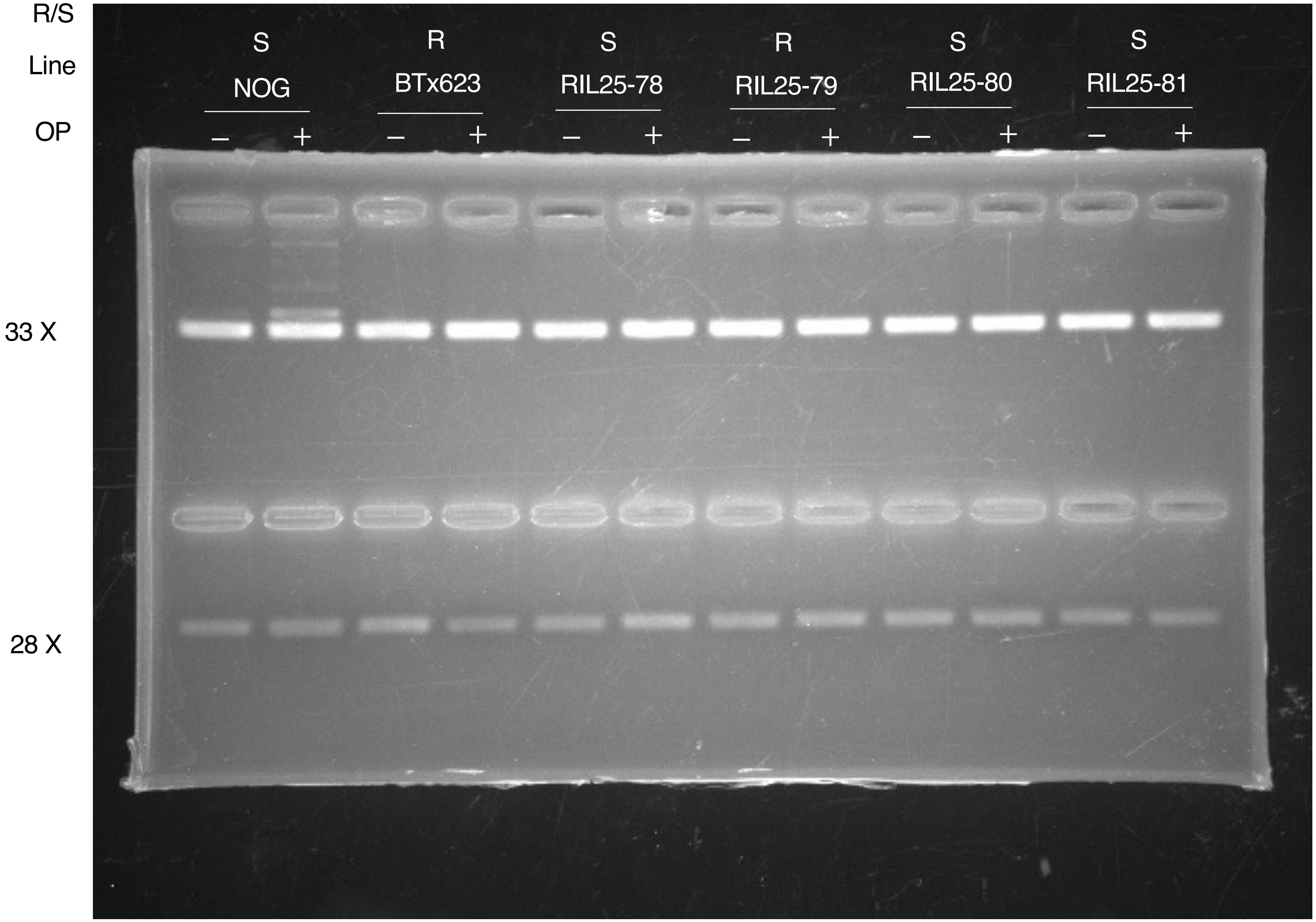

Figure 6a

NLR-C

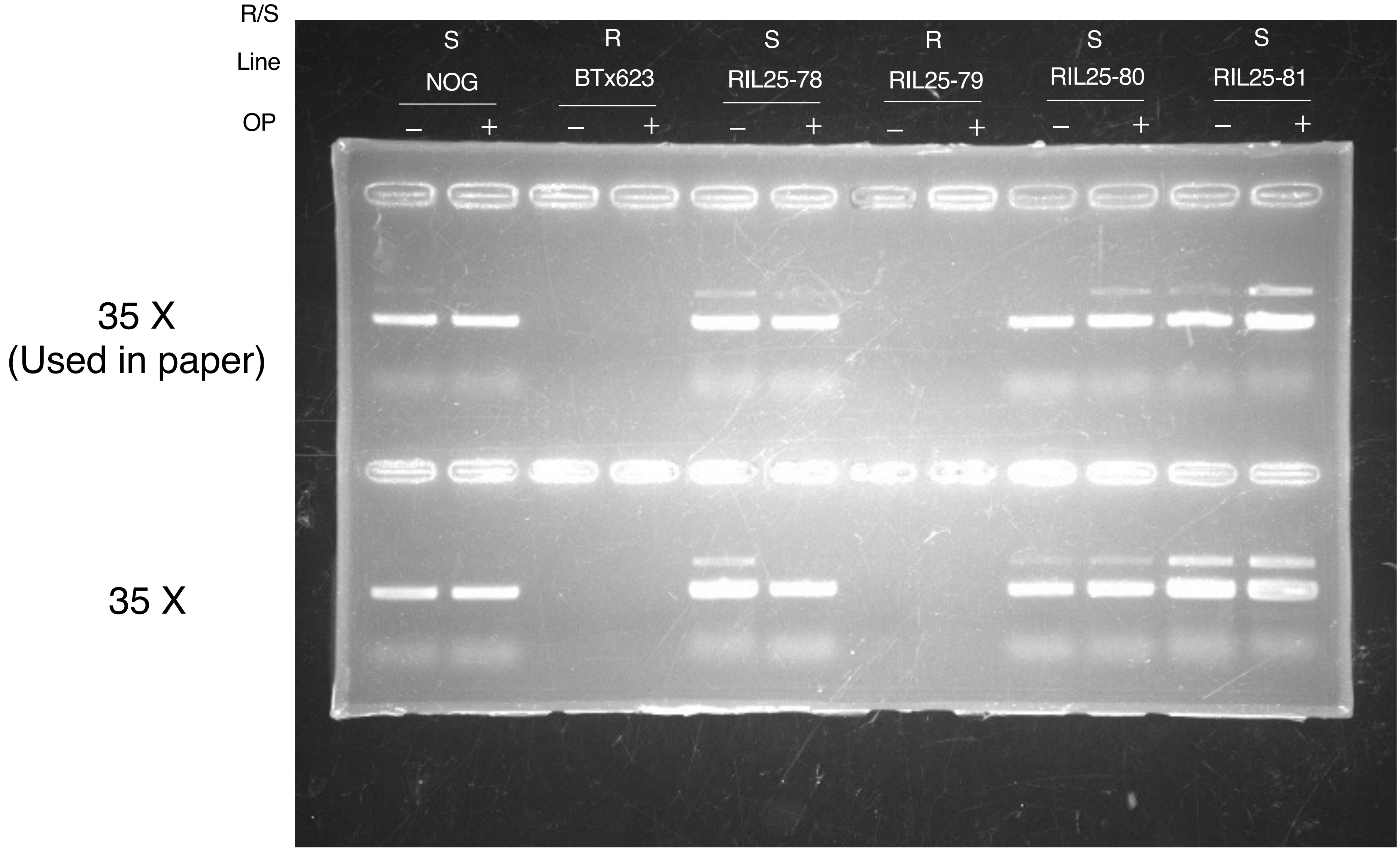

Figure 6a

NLR-C

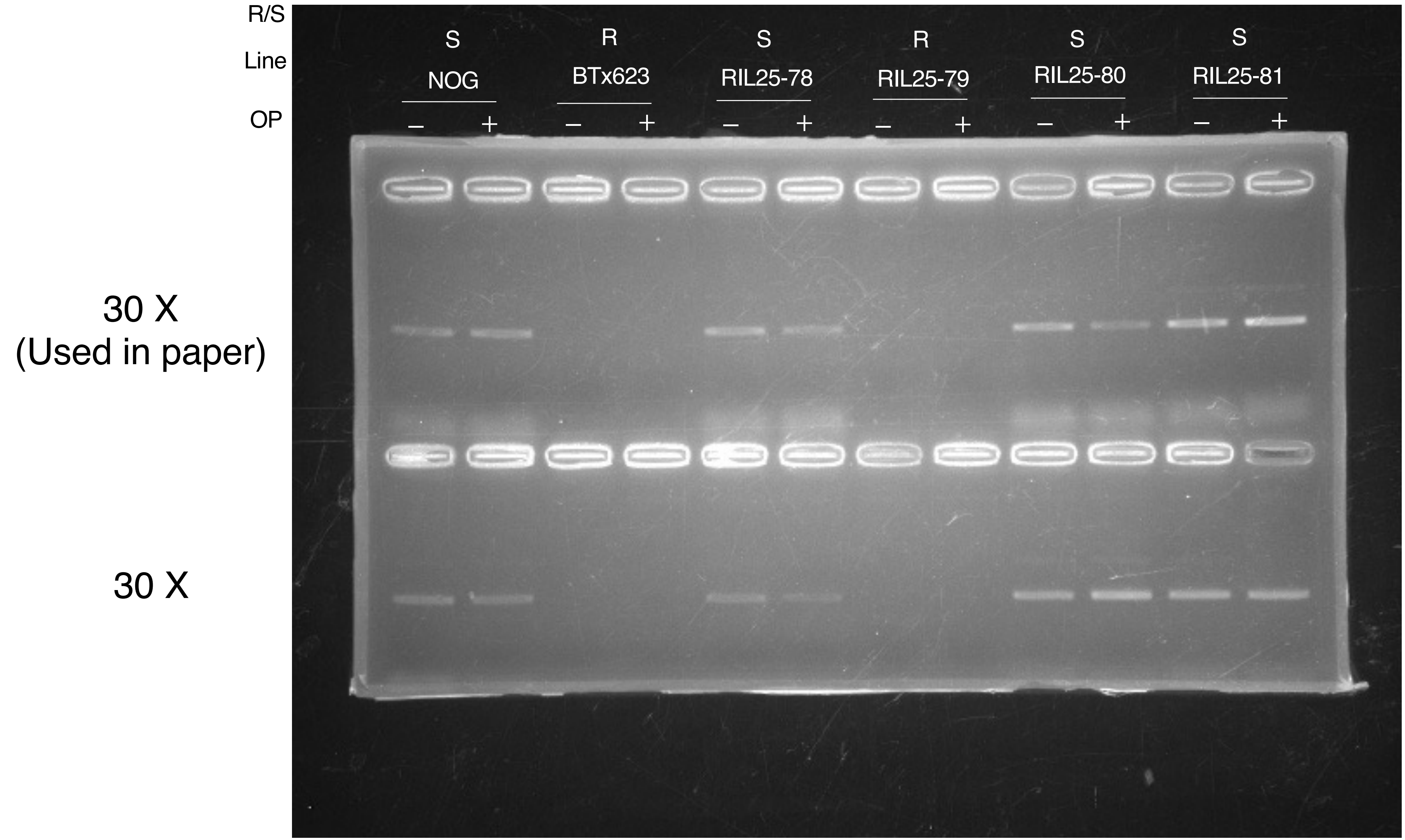

Figure 6a

NLR-B

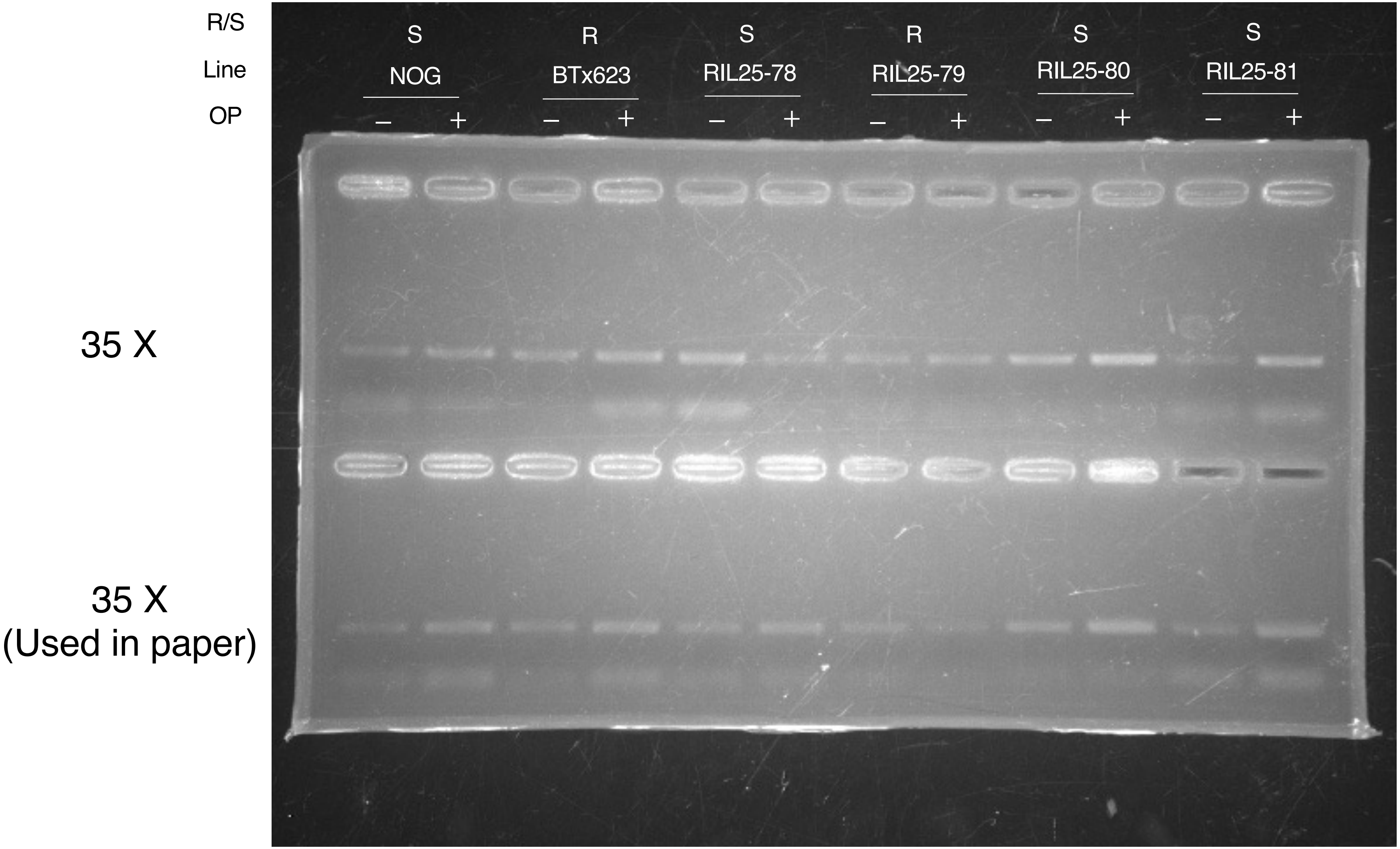

Figure 6a

NLR-B

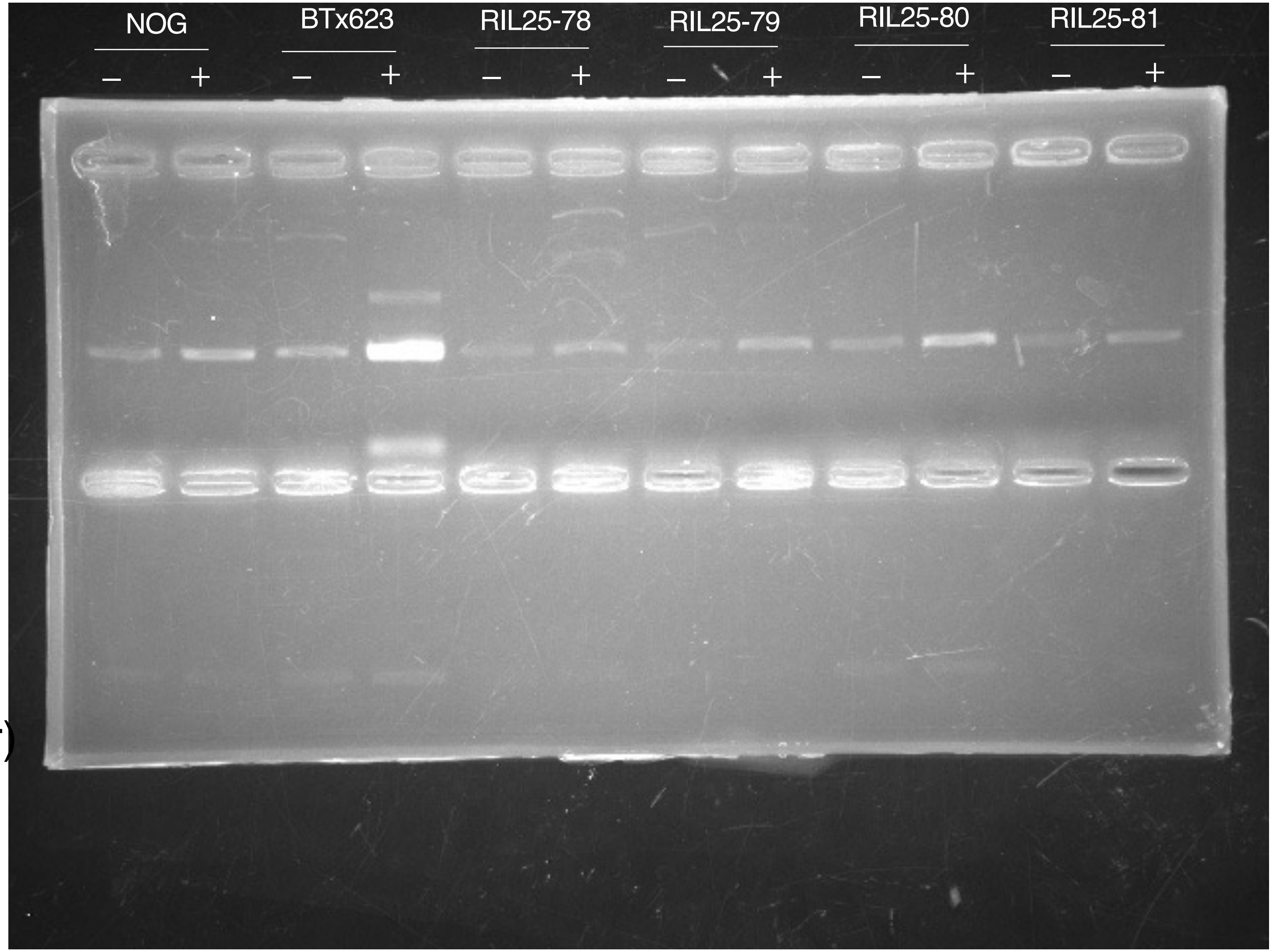

Figure 6a

NLR-A

R/S

Line

OP

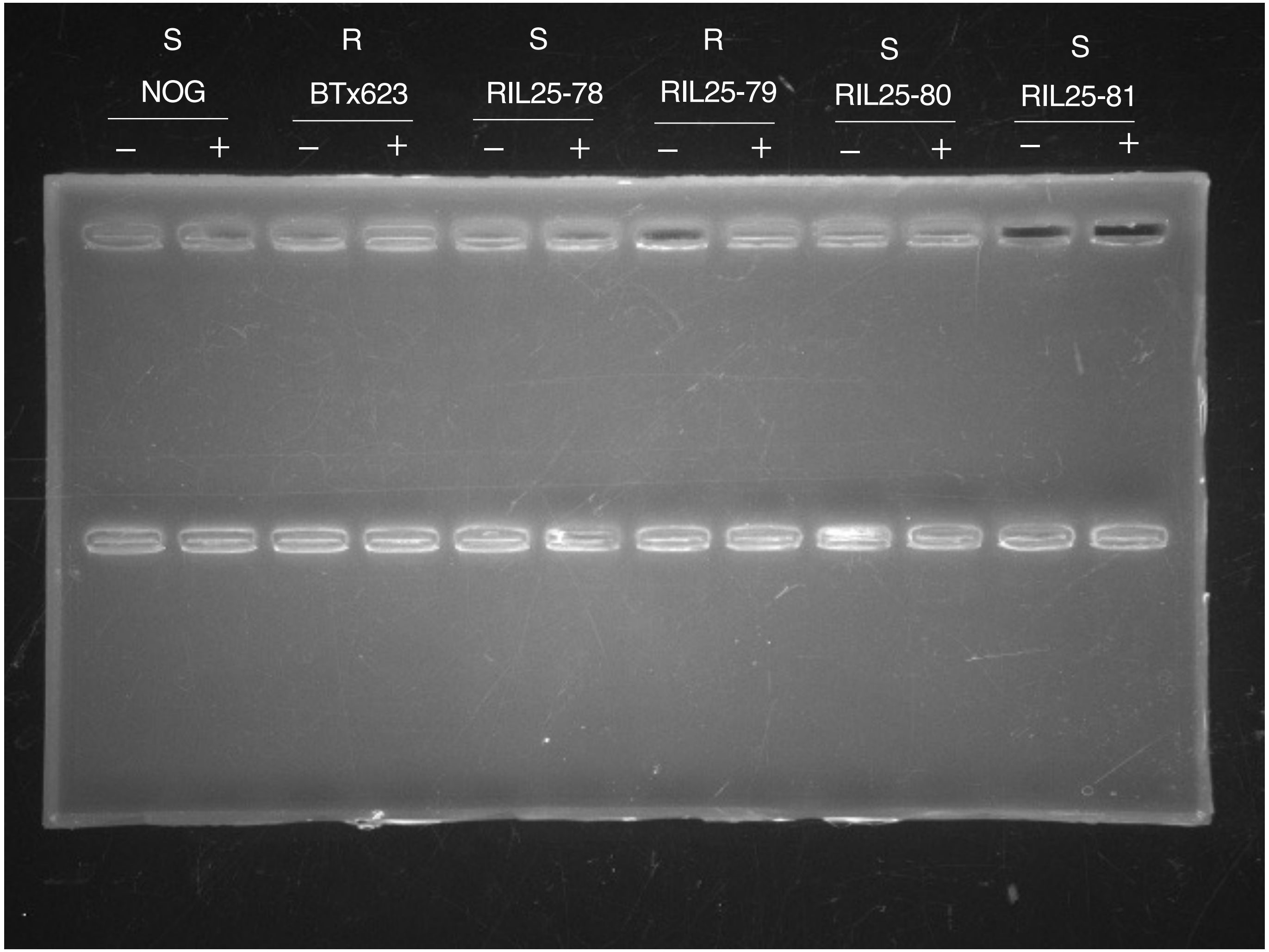

35 X
(Used in paper)

30 X
(Used in paper)

Figure S5b

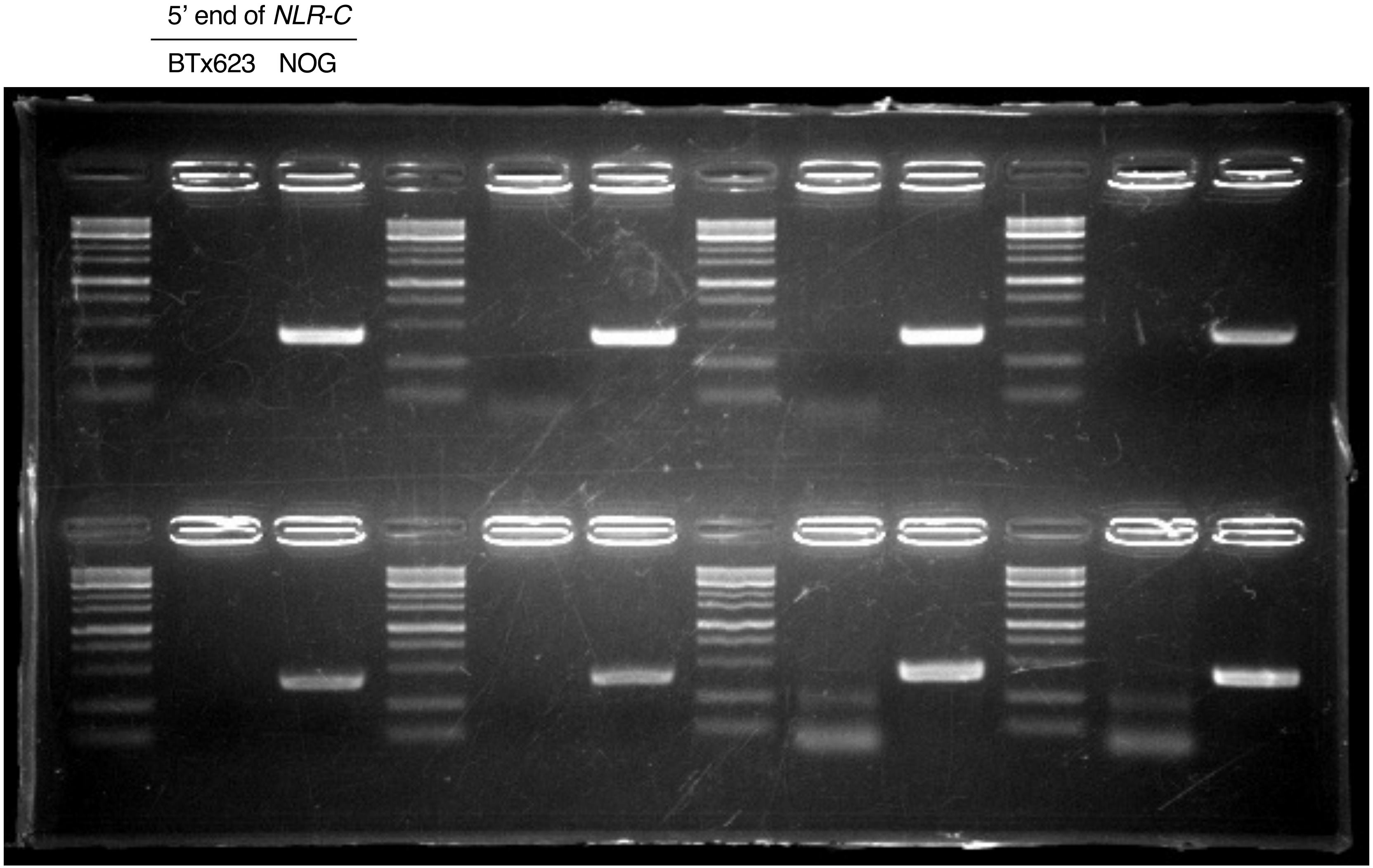

Figure S7c

NLR-A

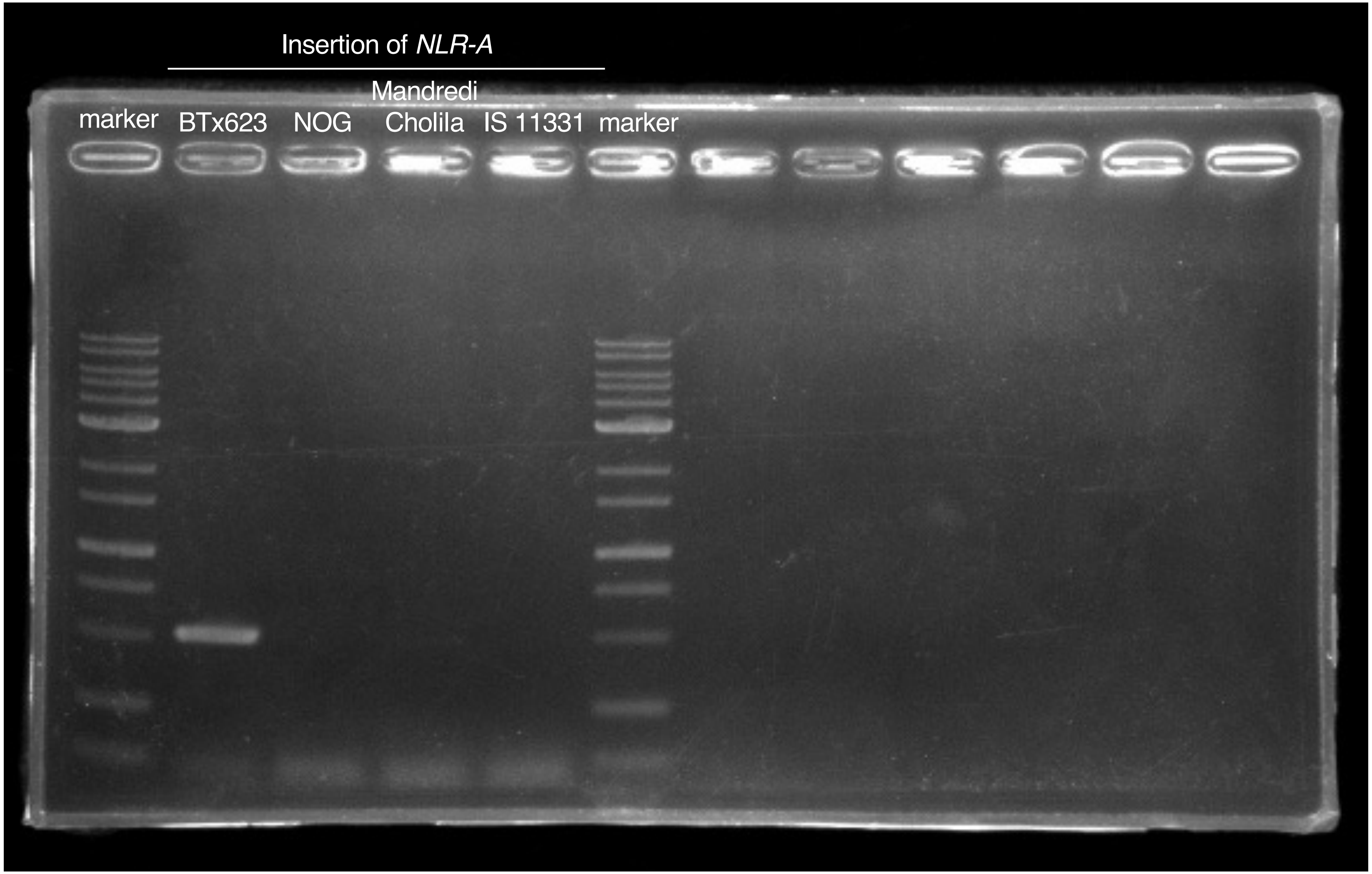

Figure S7b

NLR-C

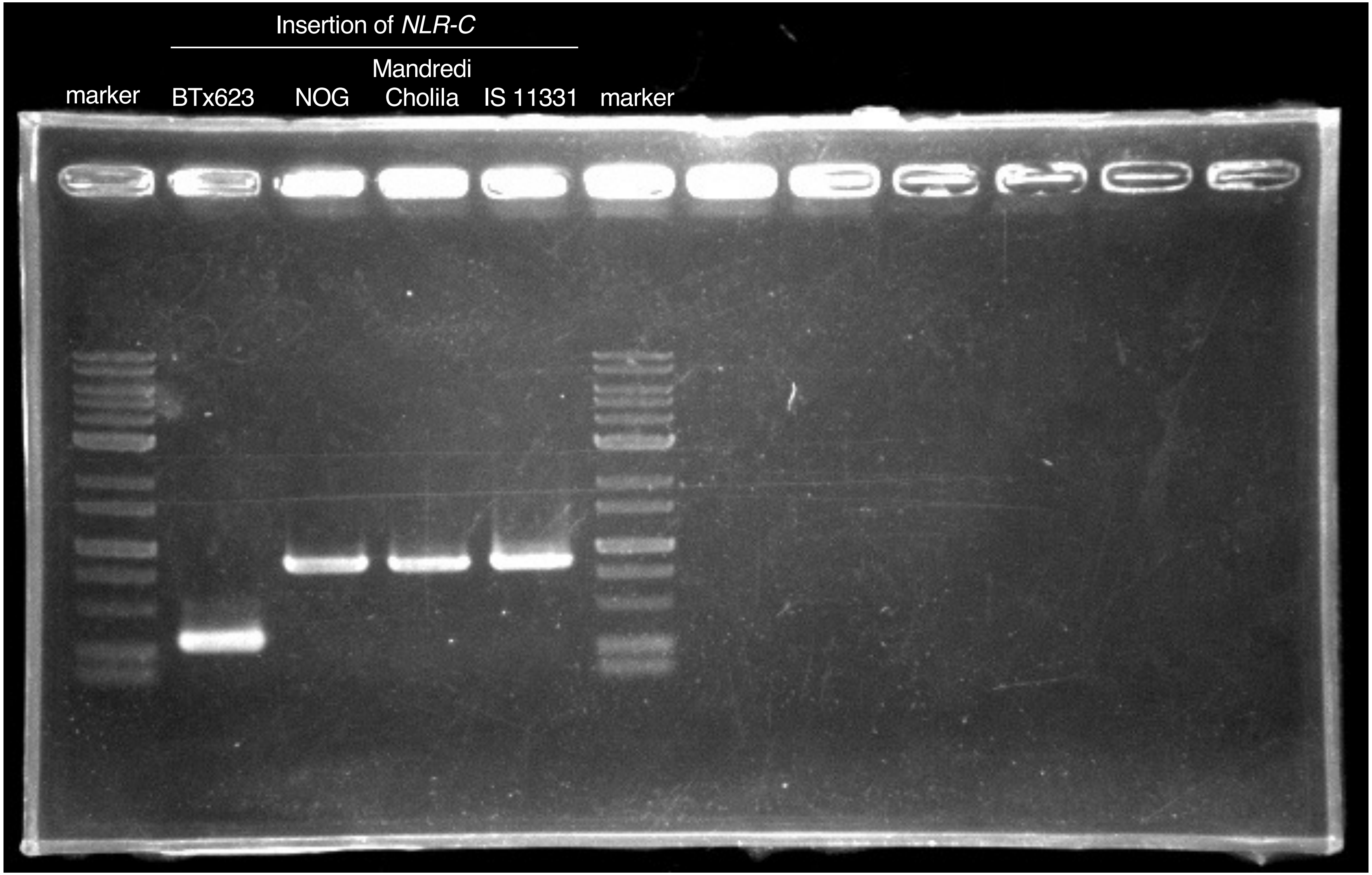

Supplement: Supplementary file 2 — Supplementary Information 2. [file 41598_2021_98908_MOESM2_ESM.pdf]
